# External and Internal Stimuli-Responsive Metallic Nanotherapeutics for Enhanced Anticancer Therapy

**DOI:** 10.3389/fmolb.2020.597634

**Published:** 2021-01-11

**Authors:** Adityanarayan Mohapatra, Saji Uthaman, In-Kyu Park

**Affiliations:** ^1^Department of Biomedical Sciences, Chonnam National University Medical School, Jeollanam-do, South Korea; ^2^Department of Polymer Science and Engineering, Chungnam National University, Daejeon, South Korea

**Keywords:** metallic nanotherapeutics, internal-stimuli, external-stimuli, phototherapy, sonodynamic therapy, magnetic hyperthermia, immunotherapy, clinical status

## Abstract

Therapeutic, diagnostic, and imaging approaches based on nanotechnology offer distinct advantages in cancer treatment. Various nanotherapeutics have been presented as potential alternatives to traditional anticancer therapies such as chemotherapy, radiotherapy, and surgical intervention. Notably, the advantage of nanotherapeutics is mainly attributable to their accumulation and targeting ability toward cancer cells, multiple drug-carrying abilities, combined therapies, and imaging approaches. To date, numerous nanoparticle formulations have been developed for anticancer therapy and among them, metallic nanotherapeutics reportedly demonstrate promising cancer therapeutic and diagnostic efficiencies owing to their dense surface functionalization ability, uniform size distribution, and shape-dependent optical responses, easy and cost-effective synthesis procedure, and multiple anti-cancer effects. Metallic nanotherapeutics can remodel the tumor microenvironment by changing unfavorable therapeutic conditions into therapeutically accessible ones with the help of different stimuli, including light, heat, ultrasound, an alternative magnetic field, redox, and reactive oxygen species. The combination of metallic nanotherapeutics with both external and internal stimuli can be used to trigger the on-demand release of therapeutic molecules, augmenting the therapeutic efficacies of anticancer therapies such as photothermal therapy, photodynamic therapy, magnetic hyperthermia, sonodynamic therapy, chemodynamic therapy, and immunotherapy. In this review, we have summarized the role of different metallic nanotherapeutics in anti-cancer therapy, as well as their combinational effects with multiple stimuli for enhanced anticancer therapy.

## Introduction

Nanotherapeutics can be the potential alternatives to standard cancer therapies such as chemotherapy, surgery, and radiation, and is an expanding sector of nanotechnology that combines nanoscience, biological science, material science, and pharmaceutical science, to develop novel anticancer therapeutics (Wang et al., 2020b). Nanoparticles (NPs) can regulate the pharmacokinetic and pharmacodynamic profiles of chemotherapeutic drugs to prolong therapeutic activity. Owing to the nanoscale size, nanoparticles take advantage of leaky tumor vasculature and defective lymphatic drainage system to enhance their accumulation and retention time inside tumors, which is mediated by the enhanced permeability and retention (EPR) effect (Martin et al., 2020). Multiple nanotherapeutics based on the EPR effect mechanism have reached clinical trials such as Doxil™, Abraxane™, Marqibo™, DaunoXome™, Onivyde™, Myocet™, Mepact™, Genexol-PM™, and SMANCS™ (Shi et al., 2017). Although most of these nanotherapeutics have only improved the solubility and pharmacokinetic profile of the anticancer drugs, few of them have improved the survival rate in clinical studies (Rosenblum et al., 2018). Passively targeted nanotherapeutics result in the non-uniform accumulation inside the tumor vasculature due to various physiological barriers like the heterogenicity of the EPR effect, variable endothelial gaps, poor tumor penetration ability, hypoxic condition, and inefficient endosomal escape (Anselmo and Mitragotri, 2016). Moreover, nanoparticles can be functionalized with different moieties and ligands to actively target the tumor through overexpressed receptor binding and enhanced targeted cell uptake (Haider et al., 2020). To enhance the therapeutic efficacy of actively targeted nanotherapeutics, it needs a broad understanding of tumor microenvironment interactions with nanotherapeutics. Classical nanomedicine incorporates two vital components, a therapeutic agent and a drug delivery carrier. Nanomedicines can accommodate chemotherapeutic drugs, nucleotides such as DNA and RNA, immunomodulatory agents, photothermal and photodynamic agents, and radioisotopes to exert potent anticancer effects (Martin et al., 2020). To develop appropriate anticancer nanotherapeutics for specific cancer types, multiple nanoparticle properties have been reported, which need to be considered and prioritized according to the therapeutic requirements. Firstly, nanotherapeutics can conquer the solubility dilemma presented by hydrophobic chemotherapeutic drugs, enhancing their systemic stability. Secondly, nano-sized drug carriers protect anticancer drugs from biodegradation or elimination through the excretory and immune systems, establishing their biocompatible nature. Thirdly, nanomedicines can be functionalized with targeting ligands and stimuli-responsive moieties for site-specific, controlled drug delivery. Moreover, premature drug release into normal tissues can be prevented, enhancing the bioavailability of nanomedicines (Ahmad et al., 2010). These characteristics need to be considered during nanomedicine formulation to magnify the therapeutic potency of anticancer drugs and avoid adverse side effects.

**Figure 1:**
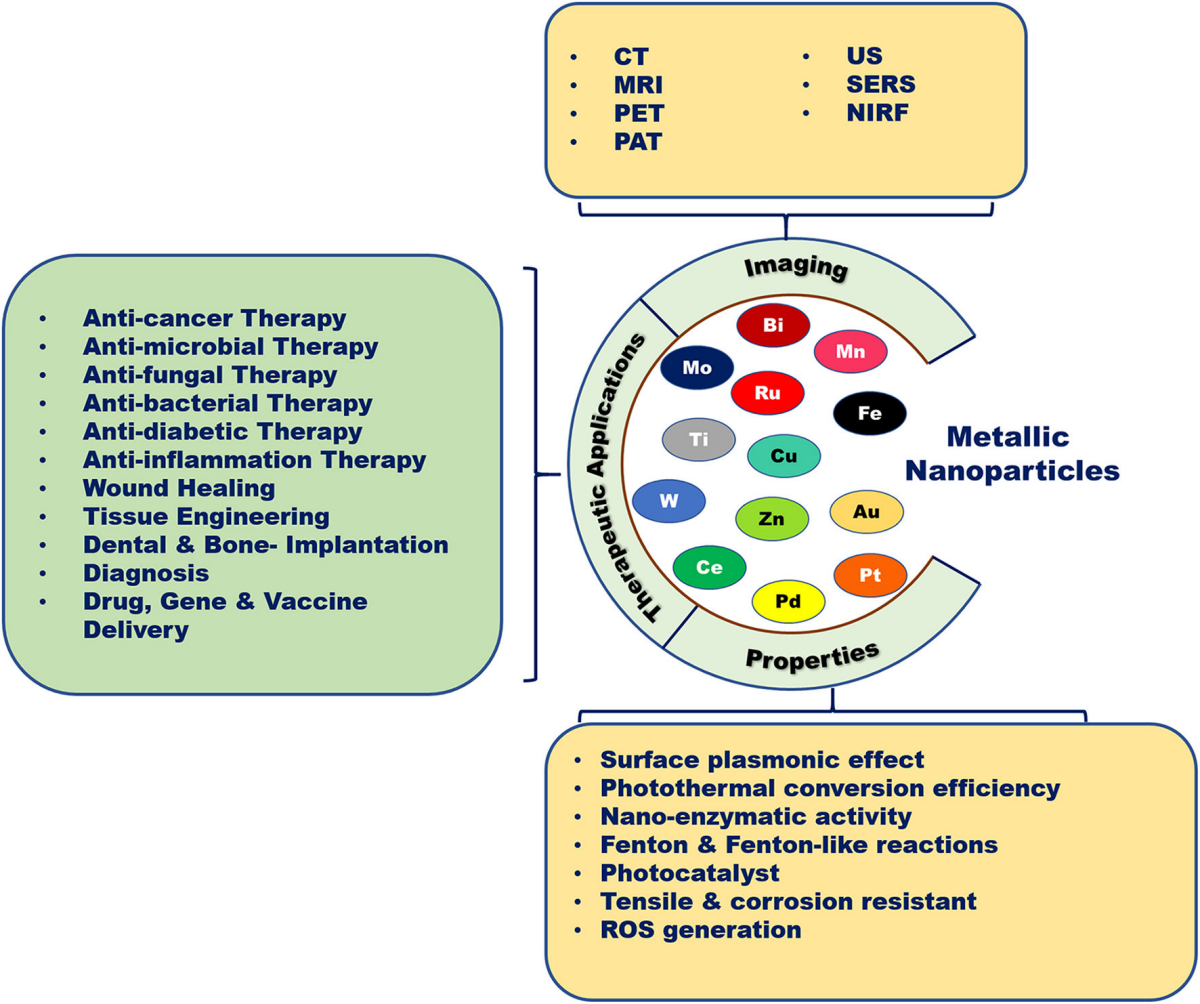
Biomedical applications of metallic nanoparticles. Metallic nanoparticles are used as potent candidates for different types of diseases due to their unique properties and imaging characteristics.

Metallic nanotherapeutics are novel multifunctional units with potential application to biomedical processes such as diagnosis, imaging, and the delivery of therapeutically active agents (Figure 1). Nanoscale metallic nanoparticles (MNPs) range in size from between 10 and 100 nanometers and can be modified during their synthesis using different strategies according to the requirements of the particular biomedical application (Mody et al., 2010). Notably, the use of MNPs is gaining attention owing to their unique thermal, magnetic, optical, catalytic, and electrical properties (Venkatesh et al., 2018). Biological fluids maintain high ionic strength, causing the destabilization and aggregation of lipids, proteins, and polymeric nanoparticles within the body (Jurašin et al., 2016). Unlike lipid-, polymeric-, and protein-based nanoparticles, MNPs can overcome stability issues in different biological environments due to their lower agglomeration tendency and facile surface functionalization (Jurašin et al., 2016). The production of MNPs is easy and cost-effective. Moreover, MNPs can be tuned into different sizes and shapes, such as nanospheres, nanorods, nanostars, nanocages, and nanotriangles, to achieve maximal therapeutic efficacy. Notably, the size and shape of MNPs can alter cellular uptake and induce immune responses against cancer (Xie et al., 2017). The high surface-to-volume ratio of metallic nanotherapeutics enables a wide range of surface functionalization with antibodies, targeting ligands, drugs, and nucleotides in cancer therapy (Conde et al., 2012). Surface modifications of MNPs can enhance cellular internalization and incorporate stealth properties against the biological milieu (Conde et al., 2012). Surface-functionalized metallic nanotherapeutics potentiate the preferential transport of anticancer drugs into cancer cells, thereby diminishing side effects to the normal tissues. MNPs facilitate imaging and diagnostic modalities, including computed tomography (CT), magnetic resonance imaging (MRI), positron emission tomography (PET), photoacoustic imaging (PA), ultrasound (US), and surface-enhanced Raman scattering (SERS) (Table 1; Sharma et al., 2018).

**Table 1:** General properties and therapeutic applications of metallic nanotherapeutics.

**Metal**	**Types of nanoparticle**	**Properties**	**Therapeutic outcome**	**Imaging**	**References**
Iron (Fe)	Fe_2_O_3_, Fe_3_O_4_	High magnetization, negative MRI contrast, nano-enzymatic activities, Fenton reaction	Hyperthermia, drug delivery, biosensing of biological molecules, chemodynamic therapy, antimicrobial activity	MRI, CT, PET	Pham et al., 2013, 2017; Cortajarena et al., 2014; Arakha et al., 2015; Shen et al., 2018; Piehler et al., 2020
Gold (Au)	Rod, star, shell, triangle, spherical-shaped Au NPs	Surface plasmon resonance effect, high electrical and heat conductivity, radioactivity and high X-ray absorption coefficient, photothermal conversion, catalytic activity, photosensitization	Plasmonic biosensing, diagnosis, photothermal therapy, photodynamic therapy, sonodynamic therapy, drug, and gene delivery	CT, PET, SERS, NIRF, PAT	Xie et al., 2017; Singh et al., 2018; Park et al., 2019; Shanei and Akbari-Zadeh, 2019; Xuan et al., 2019; Kang et al., 2020
Copper (Cu)	CuO, CuS, CuI	Magnetic properties, electrical conductivity, catalytic activity, photothermal conversion efficiency	Radiotherapy, photothermal therapy, photodynamic therapy, Fenton-like reaction, antimicrobial, antifungal therapy, wound healing, biosensing of glucose, peroxidase, antigen, and biomolecules	PET, PAT, NIRF, MRI	Hessel et al., 2011; Pham et al., 2013; Ma et al., 2018; Jiang et al., 2019b;Tao et al., 2019; Sun et al., 2020b
Bismuth (Bi)	Bi_2_O_3_, Bi_2_Se_3_, Bi2S3, BFO, Bi2Te3, BiPO4	Large X-ray attenuation coefficient, high K-edge value, chemically stable compound in biological environments, ROS production, ATP depletion	Radiotherapy, photothermal therapy, photodynamic therapy, biosensing, antimicrobial therapy, tissue engineering and implantation	CT, MRI, infrared thermography, PA, ultrasonography	Chen et al., 2019b; Badrigilan et al., 2020; El-Batal et al., 2020; Feng et al., 2020; Shahbazi et al., 2020
Platinum (Pt)	Pt-NP	Excellent catalytic agent used in various chemical reactions, platinum-catalyzed hydrogenation reactions are required for fat and vitamin production, platinum interferes with oxidation reactions used for the industrial production of organic acids, surface plasmonic resonance activity, radical scavenger, and peroxidase dismutation	Chemotherapy, photothermal therapy, radiotherapy, antibacterial and antifungal activity, cosmetic production, anti-inflammation effects, Diagnosis of tumor markers, glucose, peroxidase, proteins, and bacteria	CT	Spain et al., 2016; Barman et al., 2018; Samadi et al., 2018; Jiang et al., 2019a; Li et al., 2019d; Eramabadi et al., 2020; Fu et al., 2020
Palladium (Pd)	Pd-NP. Pd-nanosheet,	Thermal stability, photothermal stability, photocatalytic activity, optical properties, electric conductivity, ROS generator, Pro-drug activation in the TME	Chemodynamic therapy, photothermal therapy, drug and gene delivery, biosensing, anti-bacterial therapy, wound healing	PA	Dumas and Couvreur, 2015; Yan et al., 2018; Sivamaruthi et al., 2019; Yang et al., 2019c; Jiang et al., 2020; Murugesan et al., 2020; Phan et al., 2020
Molybdenum (Mo)	MoS_2_, MoO_2_, MoO	Localized surface plasmon resonance, photothermal conversion efficacy, catalytic activity, optical properties, good conductivity	Photothermal therapy, peroxidase scavenging, biosensing	PA, NIRF, CT	Liu et al., 2018; Zhan et al., 2018; Li et al., 2019a, 2020a; Sun et al., 2019
Manganese (Mn)	MnO_2_, Mn_3_O_4_, MnCO_3_, Mn-sheet, MnO_x_	Excellent catalytic activities, fluorescence quencher, T1-contrast, paramagnetic properties, pH and GSH-responsive disintegration, photothermal conversion efficiency	Chemodynamic therapy, photothermal and photodynamic therapy, radiotherapy, sonodynamic therapy, drug and gene delivery	MRI, PA, US	Casula et al., 2016; Cho et al., 2017; Wu et al., 2019b; Zhang and Ji, 2019; Chen et al., 2020; Gorgizadeh et al., 2020; Gupta and Sharma, 2020; Zhou et al., 2020b
Cerium(Ce)	Ce-NP, CeO	Strong X-ray attenuation, intracellular ROS generation, higher interconversion rate (Augustine et al., 2019; Kobyliak et al., 2019; Li et al., 2020b; Naha et al., 2020; Shin and Sung, 2020) of Ce3+/Ce4+, SOD mimetic activity, pH-sensitive pro-oxidant activity, attenuation of the pro-inflammatory cytokines and NF-kB transcription factor expression, nitric oxide scavenging	Anti-inflammation, anti-diabetic, anti-cancer, drug/gene delivery, antibacterial activity, tissue regeneration, ocular oncology	MRI, CT	Celardo et al., 2011; Dhall and Self, 2018; Inbaraj and Chen, 2019; Jia et al., 2019; Abuid et al., 2020
Ruthenium (Ru)	RuNP	Catalytic activity, luminescent property, photothermal conversion efficiency, antioxidant activity	Anti-inflammation, photothermal therapy, biosensing, antibacterial activity	Fluorescence imaging	Liu et al., 2019c, 2020; Xu et al., 2019a; Jayakumar et al., 2020
Tungsten (W)	WO_3_, WO_3−x_, M_x_WO3	Local SPR, suitable for multiple doping, strong electrical conductivity, higher X-ray absorption coefficient, pyroelectricity properties, NIR-shielding, photocatalyst, water oxidation, carbon dioxide reduction	Photothermal therapy, photodynamic therapy, anti-bacterial therapy, antimicrobial activity	CT, PET	Zhou et al., 2014; Duan et al., 2019; Levin et al., 2019; Wu et al., 2019a; Matharu et al., 2020
Titanium (Ti)	TiO_2_, TiO_1+x_	Photocatalytic activity, high tensile strength, high corrosion resistance, biological environment resistant	Antibacterial activity, tissue engineering, dental and bone implantation, drug delivery, sonodynamic therapy, photodynamic therapy	CT	Bogdan et al., 2017; Wang et al., 2017, 2020d; Azzawi et al., 2018; Çeşmeli and Biray Avci, 2019; Thomas and Kwan, 2019; Kim et al., 2020

Typically, anticancer prodrugs are designed with a specific balance of hydrophilic and lipophilic moieties (Zhang et al., 2017). After administration, the prodrugs are rapidly distributed throughout the body, irrespective of targeted and non-targeted tissues, followed by fast metabolization and subsequent excretion through the liver and kidneys. For example, liposomal and polymeric nanoparticles tend to initially accumulate in the liver, followed by excretion through the reticuloendothelial system (RES). Therefore, elaborate modifications in size, shape, and surface functionalization on nanoparticles can be beneficial in cancer therapeutics for controlling nanoparticle escape from the mononuclear phagocytic system (MPS) and enhancing circulation time. Thus, the rational design of metallic nanotherapeutics should primarily consider the biological barriers encountered during systemic circulation and the pharmacokinetic profiles of the nanotherapeutic. The surface functionalization of MNPs using hydrophilic polymers enhances their solubility and protects them from the MPS, extending the blood-retention time (Mohapatra et al., 2019). For example, passive targeting using polyethylene glycol (PEG)-coated gold nanorods significantly increased the retention time in the systemic circulation up to 19 h compared to non-PEGylated particles, which underwent rapid clearance within 15 min after administration (Lankveld et al., 2011). For metallic nanotherapeutics, active targeting involves communication between receptors overexpressed by cancer cells and a ligand conjugated on the surface of the MNPs. Hence, targeting ligand modifications on the surface of MNPs should be considered to establish receptor-mediated cellular internalization, which can enhance tumor targeting as well as therapeutic activities. However, all the reported experiments based on metallic nanotherapeutics have investigated in small animal models and laboratory experiments only which is not comparable against established traditional therapies but, expanding sector of metallic nanotherapeutics is a future leading technology and it can compete after successful clinical trials.

**Figure 2:**
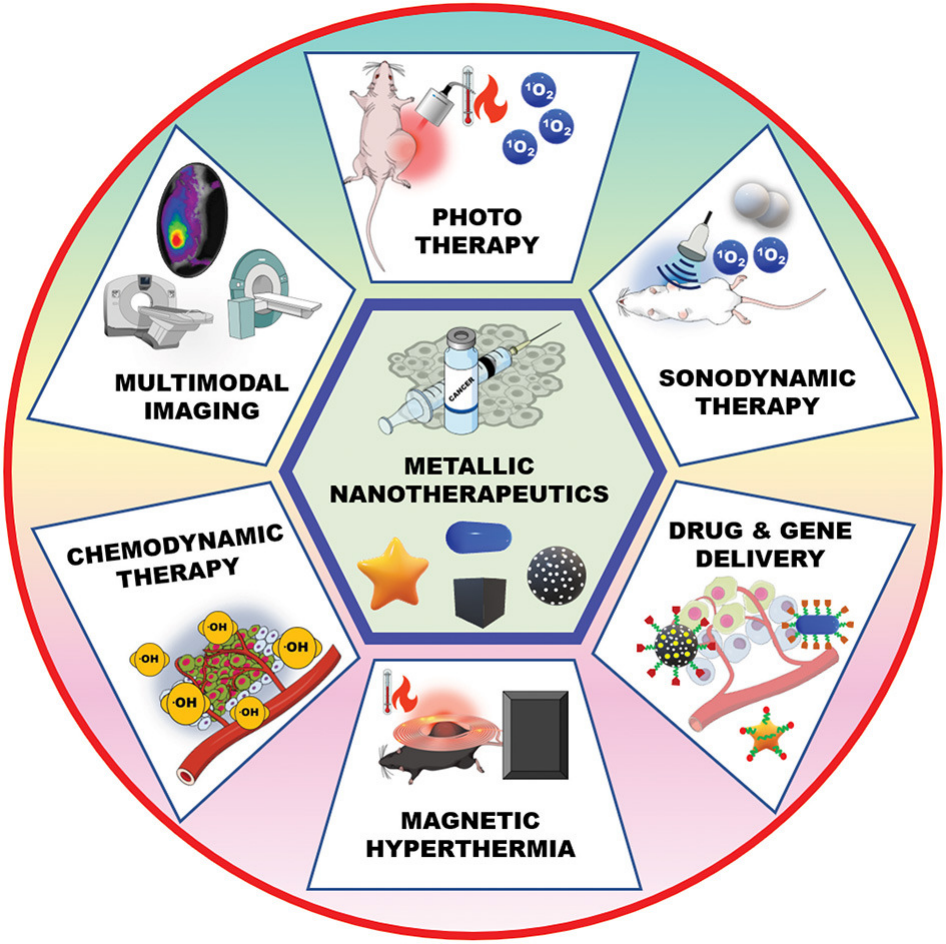
Metallic nanotherapeutic applications in cancer theranostics. Metallic nanotherapeutics are used for different types of anticancer therapies and multimodal imaging.

Metallic nanotherapeutics are termed as theranostic mediators owing to their dual applications for therapeutic and imaging purposes (Figure 2) (Silva et al., 2019). For example, light-irradiated gold nanoparticles have used in both photothermal treatment as well as CT and PA dual imaging (Xuan et al., 2019). Similarly, superparamagnetic iron oxide nanoparticles (SPIONs) have established as excellent MRI contrast agents and these are used for radiotherapy sensitization and hyperthermia treatment (Winter et al., 2020). MNPs can be combined with various stimuli, including light, an alternative magnetic field (AMF), and US, to potentiate anticancer therapeutic efficacy. Stimuli-triggered metallic nanotherapeutics have been utilized in various cancer therapies such as photothermal therapy (PTT), photodynamic therapy (PDT), magnetic hyperthermia (MHT), sonodynamic therapy (SDT), and chemodynamic therapy (CDT) to eradicate tumor tissues (Huang et al., 2020a). These therapies are associated with multiple immunogenic responses against cancer cells by inducing immunogenic cell death (ICD) and releasing tumor antigens. The release of tumor antigens into the tumor microenvironment (TME) by tumor remodeling whereby cold tumors are changed to hot tumors, modulates the infiltration of cytotoxic T cells (Huang et al., 2020a). The TME is biologically abnormal, with lower pH, hypoxia, and higher lactate and glutathione levels, which hinder therapeutic outcomes. Modulating the hypoxic and redox levels of tumors using different types of MNPs can enhance the therapeutic action. The TME can be suppressed by various tumor-suppressive agents, including myeloid-derived suppressor cells (MDSC), M2 tumor-associated macrophages (TAMs), and regulatory T cells (Tregs) within the tumor region, suppressing immune activity against cancer (Yang et al., 2017). A combination of therapies that include metallic nanotherapeutics can manipulate tumor-suppressive agents and potentiate immune activities against cancer. In this review, we detailed the stimuli that can trigger metallic nanotherapeutics to deregulate immune barriers, like MDSCs, TAMs, and Tregs, as well as combinational treatments for cancer theranostics.

## General Overview of Internal and External Stimuli

The mechanism of stimuli-triggered cancer therapeutics is based on the function of nanomaterials modulated by stimuli from outside and within the tumor. After administration, nanoparticles accumulate inside the tumor either via passive targeting through the EPR effect and leaky vasculature or active targeting with targeting moieties functionalization. Drug delivery or other therapeutic programs can be activated by specific or multiple stimuli triggers (Rajendrakumar et al., 2018b). Stimuli triggers can be promoted from the inner or outer part of the body. The internal stimuli include pH, redox, hypoxia, and enzymes, and the external stimuli include light, temperature, AMF, and US (Rajendrakumar et al., 2018b). Internal stimuli are local stimuli that are present inside the TME, whereas external stimuli are externally applied to trigger the therapeutic modality.

Internal stimulants are widely used for safe and efficient drug delivery applications in cancer therapies. Firstly, the difference in pH between healthy and tumor cells is an important aspect for a controlled drug delivery system. Tumor tissues require a high amount of energy for cellular growth, which involves increasing glucose catabolism and the concentration of lactate and hydrogen ions. Hence, the TME becomes more acidic, with a pH of 6.5 or less (Wu et al., 2018). These pH differences are extensively used in cancer drug delivery applications owing to site-specific and controllable release features (Wu et al., 2018). Secondly, glutathione (GSH) plays a significant role in cellular growth, and its concentration is higher in the cytosol and nucleus than in the intracellular and extracellular fluids. Thus, it protects cells and hinders cellular apoptosis by oxidizing reactive oxygen species (ROS) (Li et al., 2019b). Moreover, higher concentrations of GSH are detected (2–20 mM) in cancer cells compared to healthy cells which is responsible for tumor growth (Li et al., 2019b). Hence, these remarkable variations in redox concentrations promote redox-responsive nanocarriers to deliver cargoes to the targeted tumor sites (Cherukula et al., 2018). Redox-responsive linkages such as disulfide and diselenide are widely used for designing smart nano-drugs. Tumor cell-secreted enzymes, including matrix metalloproteinase (MMP) and other proteolytic enzymes, are responsible for cancer cell proliferation by triggering metabolic activity and tumor metastasis (Zhou et al., 2018). Targeting these dysregulated enzymes can enhance therapeutic activity. Enzymes are an ideal substitute for catalyzing chemical reactions under mild environments, including low temperature and neutral pH and promote biological triggers for drug release. The integration of enzyme-triggered moieties into nanotherapeutics can accomplish efficient drug release without compromising specificity. Alternatively, ROS can play a greater stimulant role during cancer nanotherapeutics. Peroxide (H_2_O_2_) is an abundant ROS present in cancer cells, promoting cancer cell proliferation, angiogenesis, apoptotic resistance, and tumor metastasis (Lopez-Lázaro, 2007). H_2_O_2_ can induce hypoxia-inducible factor- 1 (HIF-1) and genetic alternations, which are hallmarks of cancer cell invasion and metastasis (Lopez-Lázaro, 2007). Several investigations have confirmed that cancer cells possess high levels of H_2_O_2_ compared to normal cells (Revuri et al., 2019). Hence, ROS-responsive nanotherapeutics can scavenge peroxide and promote drug delivery. Manganese oxide, cerium oxide, and other MNPs are the best examples for possessing H_2_O_2−_scavenging activity and are additionally used for imaging during cancer treatment (Revuri et al., 2019; Abuid et al., 2020).

Compared to internal stimuli, external stimuli are more suitable in the field of drug delivery and cancer therapeutics. The TME varies depending upon the patient, types of cancer and pathological conditions (Wang et al., 2014). External stimuli are more feasible in controlled and disciplined manner during treatment (Wang et al., 2014) and can be manually controlled and modulated based on individual requirements. Externally stimuli triggered nanoparticles provide upgraded site-specific drug delivery, as well as constant and rapid payload release (Yao et al., 2016). However, external triggering systems necessitate the utilization of several types of specialized equipment and techniques to achieve specific stimulations. Various external stimulants was applied in cancer nanotherapeutics, including light, temperature, AMF, and US (Yao et al., 2016). AMF-induced hyperthermia and radiotherapy have been used extensively and demonstrated both positive and negative effects. Ultra-small iron oxide nanoparticles (IONPs) are considered as appropriate theranostic agents for MRI and anticancer therapy by hyperthermia and radiotherapy (Cherukula et al., 2019a; Pillarisetti et al., 2019). Recently, light-triggered phototherapy, which elevates the cellular temperature to kill cancer cells, was further investigated. Phototherapy significantly ablated tumors, inducing ICD, and reactivating cytotoxic immune cells against cancer (Rajendrakumar et al., 2018a; Cherukula et al., 2019a,b). Additionally, ultrasonic waves have been used to generate ROS and microbubbles, which interfere with cellular reactions and induce cancer cell death (Thomas and Kwan, 2019). Reportedly, various MNPS can be stimulated by both external and internal stimuli for advanced cancer therapy (Table 2). Herein, we discuss different stimuli trigger MNPs those are used in cancer therapy.

**Table 2:** Stimuli-triggered metallic nanotherapeutics for multiple anticancer therapies.

**Types of metal**	**Metallic nanotherapeutics**	**Stimuli**	**Applications**	**References**
Gold	AuNSs@PDA-PEG	NIR Laser (808 nm, 0.9 W/cm^2^)	Photothermal therapy, apoptosis, and autophagy	Li et al., 2019c
	GNR@Mem	NIR laser (980 nm, 0.5 W cm^−2^) and X-Ray (4Gy)	Photothermal therapy and radiosensitization induced apoptosis	Sun et al., 2020a
	ANS-Mas	NIR laser (810 nm, 14 W/cm^2^)	Photothermal therapy	Kang et al., 2020
	FA-AuNPs	638 nm Laser (1.56 W cm^−2^)	Photothermal and photodynamic therapy	Guerrero-Florez et al., 2020
	FA-PEG-GNP	Ultrasound (1.8 MHz, 8 ×10^−6^ J cm^−2^)	Sonodynamic therapy	Brazzale et al., 2016
	Au-MnO JNP Ves	Ultrasound (1.0 MHz, 2.0 W cm^−2^) and Redox	Chemodynamic therapy by Fenton-like reaction and sonodynamic therapy	Lin et al., 2020
Copper	CuS@MSN-TAT-RGD	NIR Laser (980 nm, 2 W/cm^2^)	Photothermal therapy	Li et al., 2018
	CuS NPs-PEG-Mal	NIR laser (808 nm, 2 W/cm^2^)	Photothermal therapy and immunotherapy	Wang et al., 2019c
	IONF@CuS	AMF (471 kHz of frequency and 18 mT) and NIR laser (1,064 nm, 0.3 W/cm^2^)	Photothermal therapy, photodynamic therapy, and magnetic hyperthermia	Curcio et al., 2019
	Cu-Cys NPs	Redox and ROS	Chemodynamic therapy	Ma et al., 2018
Molybdenum	MoO_2_ NPs	NIR laser (980 nm laser)	Photothermal therapy	Liu et al., 2018
	MoS2-ss@SiO2	NIR laser (808 nm, 1.5 W/cm^2^), Redox	Photothermal therapy, photodynamic therapy	Li et al., 2019a
Iron	USPIONs	AMF (15.92 mT at 200 kHz)	Magnetic hyperthermia	Sánchez-Cabezas et al., 2019
	Fe_3_O_4_@HA NPs	AMF 10 kA/m, 120 kHz)	Magnetic hyperthermia	Soleymani et al., 2020
	IONF@CuS	AMF (471 kHz of frequency and 18 mT) and NIR laser (1,064 nm, 0.3 W/cm^2^)	Magnetic hyperthermia, photodynamic therapy	Curcio et al., 2019
	Fe3O4-C and RLR NPs	NIR laser (808 nm, 1.5 W/cm^2^), Redox, and ROS	Photothermal therapy, chemodynamic therapy	Wang et al., 2019d
	BSO-FeS_2_ NPs	NIR laser (808 nm), redox	ROS generation, photothermal therapy, and chemodynamic therapy	Xiao et al., 2020
	Gd-Fe_3_O_4_	AMF (*f* = 370 kHz, amplitude 500 Oe)	Magnetic hyperthermia	Thorat et al., 2016
Titanium	PEG–TiO_1+x_	Ultrasound (40 kHz, 3.0 W/cm^2^)	Sonodynamic therapy	Wang et al., 2020d
	CCM-HMTNPs/HCQ	Ultrasound (1 W cm−2)	Ultrasound responsive drug delivery, sonodynamic therapy, and chemotherapy	Feng et al., 2019
	Nd:UCNPs@H-TiO_2_	NIR laser (808 nm, 4.7 W/cm^2^)	Photothermal therapy	Hou et al., 2019b
	BTiO_2_-COS	NIR laser (808 nm, 1.5 W/cm^2^)	Photothermal therapy, macrophage polarization, and immunotherapy	Zhang et al., 2019b
Palladium	FePd NPs	NIR laser (1,064 nm, 1.0 W cm^−2^)	Photothermal therapy and magnetic hyperthermia	Yang et al., 2019c
Platinum	PEG@Pt/DOX	NIR laser (808 nm, 1.5 W/cm^2^)	Photothermal therapy, NIR laser-triggered drug delivery and chemotherapy	Fu et al., 2020
Manganese	HSA-MnO_2_-Ce6 NPs	ROS, PDT laser (660 nm, 5 mW/cm^2^)	Tumor microenvironment modulation via oxygen generation and photodynamic therapy	Lin et al., 2018
	H-MnCO_3_/Ce6-PEG	ROS, PDT laser (660 nm, 5 mW/cm^2^)	Photodynamic therapy and chemodynamic therapy	Wang et al., 2019b
	Mn(ox)-LDH NPs	NIR laser (808 nm, 1 W/cm^2^)	Photothermal therapy and cell apoptosis	Xie et al., 2020
Bismuth	CD47-PEG-Bi_2_Se_3_	NIR laser (808 nm, 1 W/cm^2^)	Photothermal therapy	Guo et al., 2019
	Bi@SiO_2−_CQ	NIR laser (808 nm, 1 W/cm^2^)	Photothermal therapy	Chen et al., 2019a
	BFO NCs	Ultrasound (3 W/cm^2^, 40 kHz,), ROS	Ultrasound-triggered chemodynamic therapy	Feng et al., 2020
Cerium	UCNPs@mCeO*_*x*_*	NIR laser (980 nm laser irradiation (0.72 W cm^−2)^, ROS	Oxygen generation, photodynamic therapy, and chemodynamic therapy	Jia et al., 2019

## External Stimuli-Responsive Metallic Nanotherapeutics

### Thermo/Near-Infrared Light-Sensitive Nanoparticles

MNPs are the most effective light-sensitive vectors for temperature- elevated tumor ablation. Unlike other organic dyes and photosensitizers, MNPs are thermally stable over multiple irradiations. Following multi-ligand functionalization on the surface, MNPs have been extensively investigated for combinational therapies. Multiple metallic and bimetallic nanoparticles, metal oxides, and metal hybrids have been utilized for light-irradiated, combined PTT and PDT (Bao et al., 2016b).

#### Gold Nanoparticles

Recently, gold nanoparticles (GNPs) have gained attention because of their potential cancer theranostic applications. GNPs are potential photothermal and photodynamic transducers that may be evaluated in future clinical trials (Singh et al., 2018). Free electrons present on the surface of the GNPs are excited upon external light irradiation owing to the collective oscillation of metal conduction band electrons at a similar frequency (Kim et al., 2019). This phenomenon is termed as surface plasmon resonance (SPR). This SPR energy is transferred to the conduction band electrons, resulting in photoemission and local heating (Kim et al., 2019). The SPR property of GNPs depends on the size and shape of the nanoparticles (Huang et al., 2008). Surface plasmon absorption shifts to the near-infrared (NIR) region with increasing size or nanoparticle aggregation (Huang et al., 2008). When GNPs change from spherical to rod-shaped, the absorption band splits into a longer wavelength band in the NIR region due to longitudinal oscillation, and a shorter wavelength in the visible region, attributed to transverse oscillation (Huang et al., 2008). GNPs can be converted into different shapes such as nanorods, nanostars, nanocages, or nanoshells, which provide a wide NIR absorption range for plasmon photothermal therapy (Park et al., 2019). Gold nanostars (GNS) are a type of nanostructures possessing multiple sharp tips and have been investigated using SERS. Owing to the presence of multiple sharp tips, GNS provide tip-enhanced plasmonic properties and a wide NIR absorption range, suggesting their suitability for PTT (Park et al., 2019). Gold nanoshells have been widely investigated for NIR light-triggered PTT and PDT. Nanoshell structures have been developed using a degradable spherical template, which later forms hollow gold nanoshells (Park et al., 2019). Gold nanoshells are easy to load with surface medications and carry different types of payloads to deliver drugs upon NIR light irradiation, which promotes both cancer drug delivery and PTT. The green synthesis of GNPs using natural substrates such as citrate, chitosan, mannose, other plant-based substrates, and various biological organism-based sources improves biocompatibility and biomedical properties useful in antimicrobial and anticancer applications. Both the NIR and visible spectrum absorption of GNPs indicate several possible applications, including NIR light-triggered PTT, PDT, and imaging (Park et al., 2019).

Plasmonic nanoparticles such as GNPs can generate explosive nanobubbles upon laser irradiation which is an interesting therapeutic model for cancer therapy (Lapotko, 2011). During laser irradiation with sufficient energy, GNPs induce thermal ablation above the evaporation threshold for the NP environment which results in nanoscale boiling with surrounding medium and vapor nanobubble formation. Further, this nanobubble expands within the thin layer of the surrounding medium and collapses within a very short time. These nanobubbles generation through laser irradiation is termed as plasmonic nanobubbles (PNB). Compared to other vapor bubbles which are generated through high temperature and ultrasound irradiation, PNB can thermally insulate the outer membrane of the NPs to reduce the side effects by thermal damaging. Localization of PNB is determined in a nanoscale area surrounded by the plasmonic NPs: PNB cannot generate in an NP -free area which significantly enhances the external control and therapeutic performances (Huang et al., 2020b). Formation of PNB can be personalized by the power sources and it can be optically monetarized through different probes. PNB can be utilized for the detection and elimination of cancer cells with different combinational anticancer therapy (Huang et al., 2020b).

#### Bismuth Nanoparticles

Bismuth nanoparticles have gained momentum in cancer nanotherapeutics owing to their excellent X-ray attenuation coefficient and strong NIR absorption (Shahbazi et al., 2020). As an ideal theranostic agent, they are well-suited for CT and PA imaging and present excellent photothermal capability. Bismuth-based nanoparticles such as bismuth selenide (Bi2Se3) and bismuth sulfide (Bi2S3) are mostly used as biocompatible and cost-effective NIR agents for PTT (Chen et al., 2019b; Ding et al., 2019; Yang et al., 2019a). Bismuth rods, quantum dots, and bismuth urchins are considerably more popular agents for bimodal imaging and PTT, presenting high photothermal conversions of more than 60% (Li et al., 2017; Yang et al., 2019a). As bismuth nanoparticles lack adequate tumor-targeting capacities, various surface functionalization, cell membrane coatings, as well as cell-mediated delivery systems, have been recently investigated (Chen et al., 2019b). Platelet membrane-coated bismuth nanoparticles had prolonged circulation time and tumor-homing capability, and effective PTT was achieved (Chen et al., 2019b). Bi_2_Se_3_ nanosheet delivery with macrophage cells showed high cell targetability, whereas blank nanoparticles demonstrated poor targeting abilities (Li et al., 2017). Moreover, the high photothermal conversion efficiency presented by bismuth nanoparticles successfully eradicated tumor tissue upon NIR light irradiation.

#### Palladium Nanoparticles

Palladium nanoparticles provide excellent physiochemical properties including catalytic activities, optical properties, as well as strong thermal and chemical stability (Phan et al., 2020). Currently, palladium nanoparticles are used in dental applications, and needle-shaped palladium nanoparticles are clinically utilized for prostate and melanoma treatment (Phan et al., 2020). Typically, spherical palladium nanoparticles demonstrate poor NIR absorption efficiency and limited SPR activity. Hence, modified structures like palladium sheets and porous palladium nanoparticles have demonstrated significant photothermal abilities and have been extensively investigated (Kang et al., 2018). Moreover, ultra-thin palladium nanosheets have been developed, which produce significant heat to the tumor region upon NIR light exposure (Dumas and Couvreur, 2015). Controlled synthesis generated porous-structured palladium nanoparticles exhibiting strong NIR absorption and remarkable photothermal conversion efficiency similar to that of gold nanoparticles (Xiao et al., 2014). Furthermore, porous palladium nanoparticles exhibit superior biocompatibility compare to spherical palladium nanoparticles which have been used in several applications such as drug carriers and PTT agents (Xiao et al., 2014). Both nanosheet and porous nanoparticles have large surface areas, allowing further modifications for generating an ideal agent for cancer therapy.

#### Platinum Nanoparticles

Platinum nanoparticles are biocompatible materials, widely used because of their catalytic activity and ROS-scavenging property (Cheng and Liu, 2017). Platinum nanoparticles demonstrated strong NIR absorbance properties, emerging as prominent thermo-plasmonic light-to-heat converters (Cheng and Liu, 2017). Spherical platinum nanoparticles demonstrated photothermal efficiency similar to gold nanoshells (Wang et al., 2015a). Gold nanoparticles have been coated with platinum to potentiate NIR-irradiated PTT and ROS-scavenging (Wang et al., 2015a). Although different biosynthetic processes for platinum nanoparticles provide adequate biocompatibility, platinum ions released in the cancer cell environment induced cancer cell death and ROS generation (Cheng and Liu, 2017). However, the SPR effect of platinum nanoparticles can be achieved by treatment in the UV region, resulting in lower photothermal conversion efficiency than in other metallic nanotherapeutics (Cheng and Liu, 2017). Manikandan et al. confirmed that the modulation of nanoparticle synthesis within 1–21 nm could enhance the PTT effect, killing cancer cells (Manikandan et al., 2013). Platinum nanoparticles <6 nm in size are non-toxic but can cause cancer cell death following NIR irradiation (Manikandan et al., 2013). Typically, platinum nanoparticles are combined with other MNPs, such as Au and Fe, as a bimetallic platform to stimulate therapeutic efficacy (Samadi et al., 2018).

#### Copper Nanoparticles

Copper nanoparticles have been established as an excellent candidate for therapeutic purposes owing to their strong NIR absorption property, molar extinction coefficient, and optical imaging properties (Zha et al., 2013). Copper oxide nanoparticles are highly toxic compared to other nanoparticles as they induce greater ROS generation (Benguigui et al., 2019). The role of copper oxide nanoparticles against various cancers has been investigated in preclinical studies. However, copper selenide and copper sulfide nanoparticles demonstrated strong NIR absorbance between 800 and 900 nm. The NIR absorption property of copper nanoparticles is acquired from the d-d transition of copper ions, whereas gold demonstrates a surface plasmon effect (Li et al., 2010). Furthermore, the absorption property of copper nanoparticles, involving the d-d transitions of copper ions, differed from free-electron oscillation in the conduction band but was similar to the trapped excitation of doped metals (Li et al., 2010). Hence, the absorption range was not highly affected by the size and shape of the particles, unlike other MNPs (Li et al., 2010). Hessel et al. investigated the NIR-triggered PTT effect of copper nanoparticles that demonstrated NIR absorption with a high molar extinction efficiency and a 22% thermal transduction ability, similar to gold nanoparticles (Hessel et al., 2011). Multifunctional copper nanoparticles are highly efficient for NIR-based PTT and PA imaging for cancer theranostics.

#### Molybdenum Nanoparticles

Molybdenum nanoparticles have been widely utilized in biomedicine for imaging, therapeutic, and biosensing purposes. Primarily, molybdenum disulfide (MoS_2_) nanoparticles have been explored for theranostic approaches as 2D-nanosheets, quantum dots, and nanocages. MoS_2_ nanoparticles belong to the transition metal dichalcogenides (TMDs), demonstrating an X-M-X layered construction, where M is the transition metal (Mo, W, Ti) and X represents the chalcogenides (S, Se, Te), with atoms covalently bonded to each other within a single layer, and multiple layers attached together (Yadav et al., 2019). MoS_2_ nanostructure properties are dependent upon the arrangement of atoms and the crystallinity of the material. MoS_2_ nanoparticles have shown significant catalytic activities, photothermal conversion efficiency with an extinction coefficient of 29.2 L·mol^−1^·cm^−1^ at 800 nm laser irradiation, and multipurpose optical properties (Liu and Liu, 2018). MoS_2_ nanoparticles are reportedly suitable as NIR laser-triggered drug delivery agents that can trigger payload release upon external NIR stimuli. These NPs are easily degraded under physiological conditions. However, owing to a large surface area, these nanoparticles can be modified into a potential therapeutic model. Chen et al. reported that hyaluronic acid functionalization on MoS_2_ nanoparticles improved the stability, tumor-targeting ability, and NIR-triggered drug release, as well as application in PTT (Zhang et al., 2019a). Oxygen-deficient molybdenum oxide nanoparticles have been investigated for their strong NIR absorption property and pH-dependent degradability (Bao et al., 2016a). The intervalence charge-transfer transition between Mo elements and sufficient oxygen deficiency can cause a stronger SPR effect, resulting in a significant photothermal conversion ability (Bao et al., 2016a). Zhan et al. developed surfactant-free molybdenum oxide nanoparticles by tuning the reaction time and different phases, modulating the SPR property of the nanoparticles from the visible to the NIR range and thus, producing an adequate photothermal conversion ability (Zhan et al., 2018).

#### Tungsten Nanoparticles

Tungsten-based nanoparticles are a type of transition metal oxide exhibiting a strong localized SPR, widely used for PTT (Fernandes et al., 2020). These nanoparticles consist of perovskite units and a large band gap regulating SPR activity (Fernandes et al., 2020). Oxygen vacancy generation has been introduced in tungsten oxide nanoparticles, resulting in the alteration of the oxidation states and the formation of a new electronic state, with an appropriate number of oxygen vacancies and a narrow band gap (Wu et al., 2019a). Furthermore, the non-stochiometric property of tungsten nanoparticles is considered suitable for introducing multiple doping systems, which increases the free electrons in the conduction band (Wu et al., 2019a). However, these oxygen-deficient and doping systems can adjust the SPR activity and improve the electrical conductivity of tungsten oxide nanoparticles, resulting strong photothermal conversion efficiency. Tungsten nanoparticles exhibit a higher X-ray absorption coefficient (4.438 cm^2^/kg at 100 keV) than iodine, allowing theranostic applications of tungsten nanoparticles (Zhou et al., 2014). Zhiguo et al. developed a tungsten oxide nanorod (WO2.9) 13 nm in length and 4 nm in width. Further surface modification of WO2.9 by PEGylation enhanced the biocompatibility and potential for cancer PTT and CT imaging (Zhou et al., 2014). The synthesis of tungsten oxide nanoparticles is mostly based on a high-temperature pyrolysis process. Upon NIR laser (980 nm) irradiation, WO2.9 nanoparticles exhibited significant photothermal efficiency owing to a strong SPR effect (Zhou et al., 2014).

### Alternative Magnetic Field (AMF)-Responsive Nanoparticles

Magnetic nanoparticles (MNPs) have gained momentum in biomedical applications due to their strong diagnostic capability in MRI imaging. MNPs are efficient drug delivery vehicles that can deliver therapeutic moieties to tumors through passive, active, and magnetic targeting. External AMF exposure can cause non-invasive magnetic hyperthermia, resulting in cell apoptosis and irreversible changes in tumors. The drug delivery process can be altered by AMF exposure, which can be externally controlled to maintain adequate delivery to the tumors. IONPs are the most frequently investigated magnetic models for AMF-responsive cancer therapy.

#### Iron Oxide Nanoparticles

IONPs are an appealing agent for cancer diagnosis and therapy because of their superparamagnetic behavior that enables a wide range of activities. SPIONs with a 10-nm size have been established as a potential candidate for cancer theranostic due to their inherent magnetic property, convenient synthesis, and surface fabrication with multiple biomolecules for biomedical applications. Iron oxide has been explored as an MRI contrast agent for cancer diagnosis as well as for tracking therapeutic activity within the body. The biodegradability of IONPs has drawn massive attention because iron degraded from IONPs can accumulate as natural iron stores in the body. Several IONPs, such as Ferridex I.V.® and Ferumoxytol®, are currently in clinical use. The surface functionalization of IONPs enhanced plasma half-life and bioavailability. In comparison to other nanotherapeutics used for tumor targeting, IONPs are one step ahead as they allow passive and active targeting as well as external targeting with strong magnets. IONPs can induce local hyperthermia when exposed to AMF. AMF-induced hyperthermia can trigger cell apoptosis, protein degradation, and cell membrane destabilization, rendering cancer cells more susceptible to chemotherapy. Hence, IONPs are prominent candidates for combinational hyperthermia and chemotherapy. AMF-triggered drug delivery has an additional advantage during cancer treatment. Sami et al. reported that AMF-triggered drug release could destroy artificial 3D tumor spheroids. The macrophage-based delivery of silica nanoparticles embedded with IONPs demonstrated effective drug delivery, with the drug covalently linked to silica-coated IONPs using a thermosensitive linker. AMF triggering could significantly raise the temperature, resulting in drug release and destruction of the 3D spheroids. Combinational hyperthermia and AMF-triggered chemotherapy can induce cancer cell toxicity and avoid non-specific delivery, which is essential for cancer therapy.

#### Hybrid Ferrite Nanoparticles

Different nanosized (10–100 nm) IONPs, including magnetite (Fe_3_O_4_), maghemite (γ-Fe_2_O_3_), and hematite (α-Fe_2_O_3_), reportedly induced magnetic hyperthermia and have been utilized for MRI imaging in cancer theranostics (Can et al., 2012). Researchers have adjusted the intrinsic properties of IONPs by doping with multiple transition metal ions. A small number of zinc (Zn^2+^) substitutions in IONPs alters their magnetic properties. Zn^2+^, a diamagnetic cation possessing zero magnetic moments, can substitute for the iron cations in the tetrahedral and octahedral sites and weaken antiferromagnetic coupling, resulting in stronger magnetization saturation (Hadadian et al., 2019). Doping with an appropriate amount of Zn^2+^ strongly affected the *Curie* temperature (Tc) and hyperthermia performance of IONPs, where large amount of doping resulted in a canted spin and decreased magnetization (Hadadian et al., 2019). Furthermore, doping with various magnetic nanoparticles can reduce the size of the hybrid IONPs, which assists in immune system escapes and prolonged circulation. Similarly, doping with cobalt ions resulted in smaller-sized cobalt ferrite nanoparticles (size < 12 nm), demonstrating stronger hyperthermia efficiency and MRI contrast than IONPs of similar size (Fantechi et al., 2015). However, bare cobalt ferrite nanoparticles are toxic. Surface functionalization with biocompatible polymers and adjusting the doping content of the cobalt ions can avoid these issues and potentiate the biocompatibility of the particles without compromising performance (Fantechi et al., 2015). Ruby et al. have reported hybrid IONPs with manganese doping could enhance AMF-induced hyperthermia with T1 and T2 dual-mode MRI for anticancer therapy (Gupta and Sharma, 2020). Water-soluble and easily synthesized Mn-doped IONPs possessed similar morphology and intrinsic magnetic properties (Casula et al., 2016). Along with cobalt and manganese ions, various studies have utilized copper, nickel, bismuth, and gadolinium ions for doping IONPs to enhance MRI contrast efficiency and magnetic hyperthermia. These hybrid nanoparticles are the future of AMF-triggered cancer therapy, presenting better applications compared to single nanoparticles.

### Ultrasound (US)-Responsive Nanoparticles

US is a mechanical sound wave with a frequency >20 kHz, which is higher than the hearing range of humans. Compared to other external stimuli, US possesses a significant advantage, with stronger tissue penetration due to its non-radiative and low tissue-attenuation properties (You et al., 2016). US mediates both thermal and non-thermal effects, which can be used in treatment. Alterations in the frequency, scattering, and absorption can modulate the generation of cavitation, which renders it useful in imaging and drug-release applications. High-intensity focused US (HIFU) has been clinically established for cancer treatment as well as diagnostic purposes. Various sonosensitizers, which include both organic and inorganic materials, have been investigated in US-based cancer therapy and imaging. Reportedly, metallic sonosensitizers demonstrate greater efficacy than other sonosensitizers in generating ultrasonic cavitation-based microbubbles and toxic radicals to adequately kill cancer cells.

#### Titanium Oxide Nanoparticles

**Figure 3:**
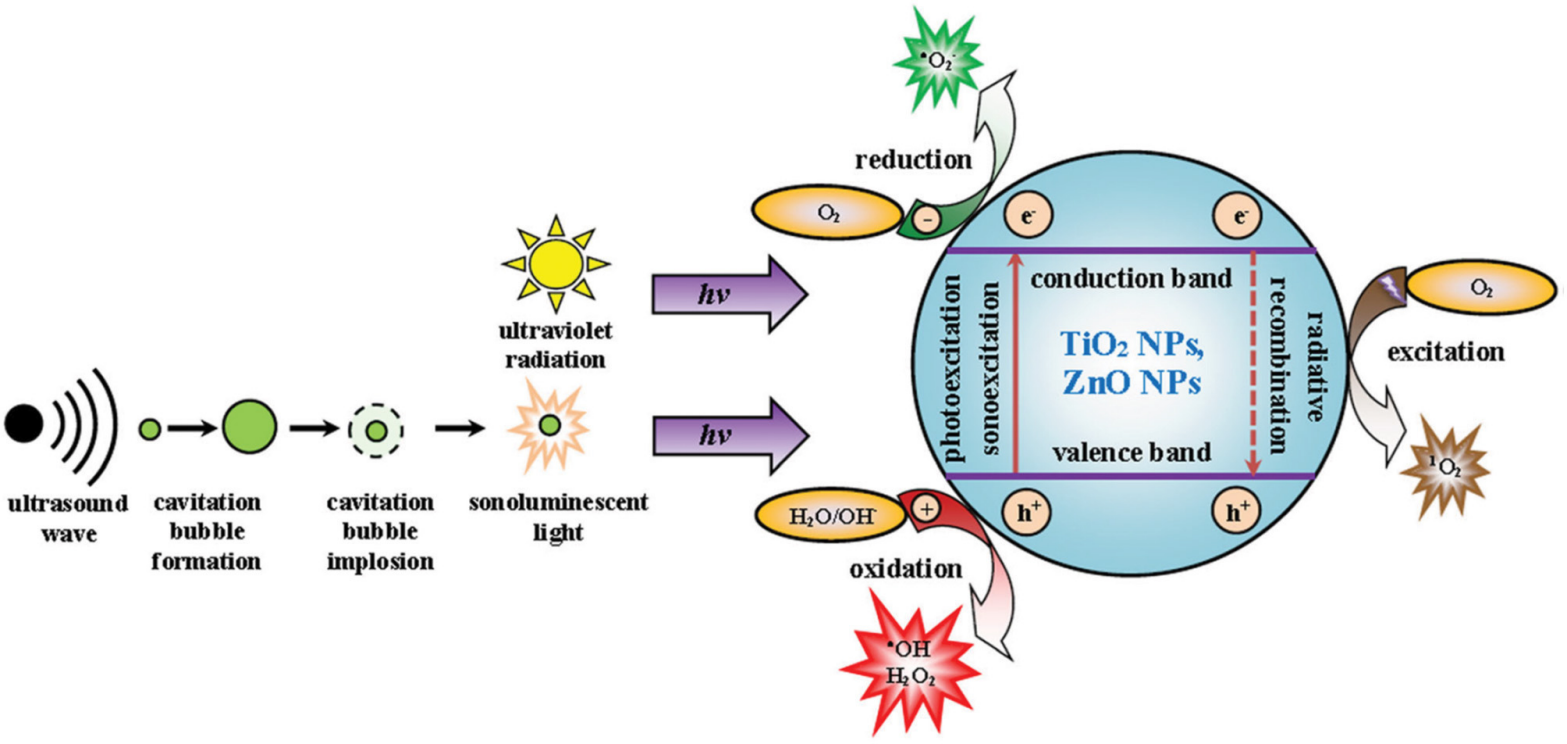
Mechanism of cavitation and ROS production by TiO_2_ and ZnO nanoparticles. Reproduced with permission (Bogdan et al., 2017) *Copyright* © *2017, Springer Nature*.

Titanium dioxide (TiO_2_) nanoparticles are chemically inert and stable in physiological environments and considered as biocompatible models for therapy (Ninomiya et al., 2012; Canavese et al., 2018). TiO_2_ nanoparticles have two different crystal structures, anatase, and rutile. Rutile TiO_2_ nanoparticles are fine-structure nanoparticles, whereas anatase possesses a crystal structure (Bogdan et al., 2017). The crystal structure allows anatase TiO_2_ NPs to generate ROS and the NPs have been used as a photocatalyst. Rutile TiO_2_ nanoparticles are fine in structure and chemically inert. However, smaller-sized TiO_2_ nanoparticles possess a larger surface and cause toxicity (Bogdan et al., 2017). The larger surface area of TiO_2_ nanoparticles, compared to that of microparticles, allows for the absorption of UV radiation, enhancing the excellent photocatalyst properties of TiO_2_ nanoparticles (Bogdan et al., 2017). The surface modification of TiO_2_ nanoparticles can modulate therapeutic behavior and systemic toxicity. TiO_2_ nanoparticles belong to the semiconductor metal oxide group, consisting of an electron-enriched valence band and an electron-free conduction band (Bogdan et al., 2017). The bandwidth gap of TiO_2_ nanoparticles is reportedly 3.20 eV and excited in the UV radiation range. During irradiation, the electron transfer from the valency bond to the conduction band creates electron excitation. Excited electrons can reduce molecular oxygen to superoxide radicals, while the positive ions oxidize water molecules to generate hydroxyl radicals and H_2_O_2_. Several investigations have reported the excitation of TiO_2_ nanoparticles under sonoexcitation in aqueous solution (Çeşmeli and Biray Avci, 2019). In aqueous conditions, sonoexcitation under US waves induces a temporary dilution from the loss of pressure, which causes the cavitation of bubbles and thickening of the liquid, resulting in the collapse of the bubbles (Figure 3). This phenomenon is termed acoustic cavitation, which acts as a sonocatalyst, generating toxic effects and emitting sonoluminescence during cavity collision (Çeşmeli and Biray Avci, 2019). Furthermore, the shape of TiO_2_ nanoparticles can affect cavitation and ROS formation during treatment. Reju et al. reported that nanocone-structured TiO_2_ nanoparticles accelerated the inertial cavitation process and enhanced cell penetration under US exposure (Thomas and Kwan, 2019). Surface-modified hydrophilic TiO_2_ nanoparticles have shown prolonged blood circulation and a high level of ROS upon US irradiation (You et al., 2016). US-triggered SDT can upregulate pro-inflammatory cytokines within the tumor and destroy the tumor microvasculature (You et al., 2016). Multiple anti-tumor therapeutic effects are the key feature of SDT, which can be utilized in future clinical applications.

#### Gold Nanoparticles

GNPs are extensively used in cancer therapy owing to their unique optical properties. GNPs are appropriate models to induce PTT and PDT effects upon laser irradiation. In tumors, laser light absorption by GNPs results in the production of vapor cavities around the nanoparticles (Shanei and Sazgarnia, 2019). This cavitation ability introduces a strong SDT effect and improves US imaging (Shanei and Sazgarnia, 2019). HIFU is a potential cancer therapy technique to target a small focused region in the body and penetrate soft tissue to induce thermal ablation (Shanei and Sazgarnia, 2019). In tumors, the surrounding liquid provides a nucleation site for GNPs to achieve acoustic cavitation and bubble formation, with the surface roughness causing bubble collisions under HIFU irradiation (Shanei and Sazgarnia, 2019). Notably, folic acid-conjugated GNPs selectively target breast cancer cells. Low-intensity US irradiation results in marked ROS production, whereas high-intensity US irradiation causes both ROS production and hyperthermia (Serpe et al., 2020). US irradiation causes thermal and mechanical effects and produces singlet oxygen and toxic hydroxyl radicals by reacting with the surrounding liquid (Serpe et al., 2020). Lin et al. developed gold-manganese oxide nanoparticles that decomposed in the TME to initiate a Fenton-like reaction and generate additional hydroxyl radicals (Lin et al., 2020). US-triggering improved the efficacy of the Fenton reaction and stimulated ROS generation (Lin et al., 2020). Ahmad et al. investigated the combination of radiosensitization and sonodynamic effects in cervical cancer cells (Shanei and Akbari-Zadeh, 2019). US irradiation enhanced the radiosensitivity of cancer cells and combination therapy increased the cytotoxic effect from low-intensity US waves by up to 95.8% (Shanei and Akbari-Zadeh, 2019).

## Internal Stimuli-Responsive Metallic Nanotherapeutics

### ROS and GSH-Responsive Nanoparticles

ROS is considered the most crucial factor associated with cellular physiological processes such as cell growth, cell proliferation, cell signaling, and pathological activities. ROS is endogenously produced during cellular respiration when oxidase receives the electrons released from membrane carriers, including ubiquinone and cytochrome c, and converts them into superoxide ions. In the cytosol and mitochondria, superoxide ions are rapidly converted to H_2_O_2_ by superoxide dismutase (SOD) and xanthine oxidase. Other functions inducing ROS generation include NADPH oxidase reactions, the β-oxidation of fatty acids, flavin oxidase reactions in the peroxisomes, and the protein oxidation process. Notably, H_2_O_2_ has a longer lifespan inside cells compared to other ROS. Oxidative stress results in H_2_O_2_ overproduction and induces pro-inflammatory cytokines and cellular apoptosis. The scavenging ability of H_2_O_2_-responsive nanomaterials is used in drug delivery to inhibit hypoxic conditions by generating oxygen. Various MNPS have demonstrated H_2_O_2_ degradation and oxygen regeneration to reduce ROS levels, hypoxic conditions, and undesirable side effects. H_2_O_2_ can stimulate the generation of toxic radicals such as hydroxyl radicals and peroxide ions to induce cellular apoptosis.

#### Manganese Oxide Nanoparticles

**Figure 4:**
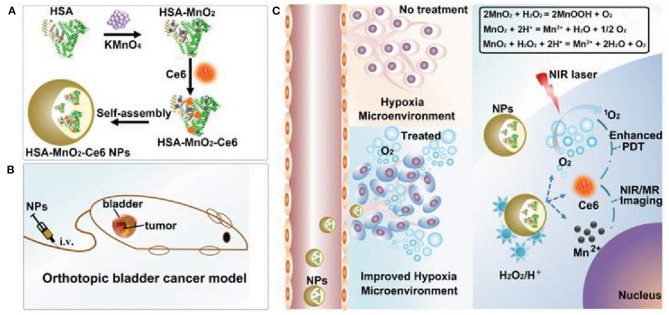
Schematic representation of self-assembled MnO_2_ nanoparticles modified with photosensitizer for photodynamic therapy (PDT) **(A)**. Application of MnO_2_ nanoparticles catalyze endogenous peroxidase to oxygen and water and enhance the PDT efficacy upon laser exposure **(B,C)**. Reproduced with permission (Lin et al., 2018) under *copyright Creative Commons Attribution 4.0 International License (CC-BY license)*.

The physical and chemical properties of manganese oxide (MnO_2_) nanoparticles have made them vital MNPs for biomedical applications. MnO_2_ nanoparticles demonstrate greater optical properties, potent oxidizing capability, and strong absorption, enhancing their utility in cancer applications (Wu et al., 2019b). MnO_2_ nanoparticle compositions such as MnO, Mn_5_O_8_, Mn_2_O_3_, Mn_3_O_4_, and MnO_2_ have been investigated in cancer therapy. Various MnO_2_ nanoparticle compositions have been synthesized using different protocols, allowing broad applications such as imaging, phototherapy, catalytic activity, and drug delivery (Wu et al., 2019b). Furthermore, MnO_2_ nanoparticles possess remarkable oxidizing properties that can improve the hypoxic tumor environment. In cancer cells, the main function of MnO_2_ nanoparticles involves interacting with endogenous H_2_O_2_ and O_2_ by reacting with reduced-state oxygen to reduce hypoxia, as well as reducing acidosis by interacting with various ions (Chen et al., 2020). Owing to the lack of oxygen inside the TME, PDT agents tend to generate fewer ROS, resulting in unsuccessful PDT. MnO_2_ nanoparticles react with TME-based H_2_O_2_ and generate Mn^+^ ions, water, and O_2_. Reoxygenation of the TME modulates the therapeutic effects as PDT laser irradiation converts the regenerated O_2_ molecule into singlet oxygen, causing cancer cell death (Chen et al., 2020). Released Mn^+^ ions are used for T1 contrast-based MRI imaging, enabling the theranostic application of MnO_2_ nanoparticles (Chen et al., 2020). The hypoxic environment of cancer cells is induced by insufficient oxygen delivery and the rapid cell proliferation of tumor tissue cells. Furthermore, hypoxia induces HIF-1 protein, which is responsible for aggressive tumor development i.e., three times more resistant to therapy (Vaupel and Mayer, 2007; Devarasetty et al., 2020). Alleviation of the hypoxic environment downregulates hypoxic markers in cells, including HIF-1α, which promotes multidrug resistance (MDR) and reoxygenates the tumor environment to promote therapeutic efficacy. Lin et al. reported that oxygen-producing HAS-MnO_2_-Ce6 nanoparticles relieved hypoxia and treated bladder cancer (Lin et al., 2018). Albumin protein-biomineralized MnO_2_ nanoparticles were shown to re-oxygenate the TME and double the PDT efficacy following PDT laser irradiation (Figure 4). Biocompatible materials modified MnO_2_ nanoparticles demonstrated excellent therapeutic activity without any side effects. Radiation therapy produces ROS (•OH) by the radiolysis of water molecules in the tumor, which can cause cancer cell death. However, high concentrations of intracellular GSH neutralize intracellular ROS to protect the cell, limiting the effects of radiotherapy (Cho et al., 2017). Reportedly, MnO_2_ nanoparticles oxidize GSH to glutathione disulfide (GSSG), reducing GSH levels in the tumor, promoting radiotherapy effects, and enhancing ROS activity against cancer cells (Cho et al., 2017). Yang et al. developed attractive hollow MnO_2_ nanoparticles for TME-specific on-demand drug delivery and imaging. The MnO_2_ nanoparticles modulated the hypoxic TME conditions and significantly improved the antitumor responses. The hollow template allows for the co-loading of MnO_2_ nanoparticles with Ce6 (photosensitizer) and doxorubicin (DOX) for synergistic anticancer effects. MnO_2_ nanoparticles reduce the hypoxic environment via the generation of molecular oxygen through intracellular H_2_O_2_ reactions. Adequate oxygen generation improved Ce6 activity, demonstrating significant ROS generation and DOX activity against cancer cells (Yang et al., 2017).

#### Cerium Oxide Nanoparticles

In recent years, cerium oxide nanoparticles have been investigated for biomedical applications. CeO_2_ nanoparticles demonstrate diverse advantages such as strong redox ability, low toxicity, and significant catalytic activity (Dhall and Self, 2018). Additionally, these nanoparticles possess a unique mixture of both Ce^3+^ and Ce^4+^ ions on their surface, where the ratio of Ce^3+^ and Ce^4+^ affects the nanoparticle size. The size of CeO_2_ nanoparticles depends on the Ce^3+^ composition. Increasing the Ce^3+^ concentration can reduce particle diameters and enhance oxygen deficiency (Dhall and Self, 2018). Additionally, these nanoparticles are incorporated with a fluorite crystalline lattice structure, resulting in a higher reactive surface area to scavenge free radicals. Owing to a lower redox potential (=1.52 v), CeO_2_ nanoparticles can shift between Ce^3+^ and Ce^4+^, depending upon the experimental conditions, a key feature for using CeO_2_ nanoparticles as an antioxidant carrier (Siposova et al., 2019). Furthermore, the redox property of CeO_2_ nanoparticles has been widely utilized against ROS-based tissue damage. A higher Ce^3+^-to-Ce^4+^ ratio allows superoxide reduction as Ce^3+^ is easily oxidized to Ce^4+^. The oxidation and reduction processes between Ce^3+^ and Ce^4+^ are reversible reactions, which spontaneously react with H_2_O_2_ to produce Ce^3+^ and molecular O_2_ (Celardo et al., 2011). The ROS-scavenging activity of CeO_2_ nanoparticles is a spontaneous, recyclable reaction. CeO_2_ nanoparticles react with superoxide ions and are reduced to H_2_O_2_ and Ce^4+^, with the regenerated Ce^4+^ ions again oxidizing H_2_O_2_ to oxygen molecules (Celardo et al., 2011). This spontaneous reaction cycle can scavenge H_2_O_2_ and superoxide simultaneously and protect from the paradoxical effect of SOD and catalase activities. Based on previous reports, lower Ce^3+^-to-Ce^4+^ ratios in CeO_2_ nanoparticles demonstrated nitric oxide radical scavenging, whereas higher ratios scavenged peroxide and toxic hydroxyl radicals (Inbaraj and Chen, 2019).

#### Iron Oxide Nanoparticles

IONPs are unique MNPs used extensively in biomedical applications and are superior theranostic agents in MRI and radiotherapy against cancer. IONPs have been widely investigated for oxidase-like behavior in the Fenton reaction as well as for peroxidase-like activity for H_2_O_2_ scavenging. During the Fenton reaction, Fe^2+^ reacts with surrounding H_2_O_2_, which is excessively produced by the aerobic processes of cancer cells, generating toxic radicals (•OH) by oxidizing Fe^2+^ to Fe^3+^ (Ranji-Burachaloo et al., 2018). The toxic radicals generated via the iron-based Fenton reaction induced non-apoptotic cell death, which was termed ferroptosis by Dixon et al. (2012). Among the various IONPs, Fe_3_O_4_ and αFe_2_O_3_ IONPs react with endogenous H_2_O_2_ in low pH conditions to advance Fenton reactions and generate (•OH) radicals and those hydroxyl radicals are highly reactive and possess biological activity (Ranji-Burachaloo et al., 2018). Hence, IONPs ar used for CDT in combination with other therapies such as PTT, PDT, and immune therapy. To overcome the limited solubility of IONPs, a photodynamic photosensitizer (Ce6) was incorporated on the surface of SPION nanoclusters (Ce6-SCs) by preparing an oil-in-water emulsion (Amirshaghaghi et al., 2019). The therapeutic performance of the Ce6-SCs was evaluated by the systemic administration to tumor-bearing mice. Hybrid IONPs produced excess O_2_ with H_2_O_2_ scavenging, which altered PDT efficacy (Amirshaghaghi et al., 2019).

#### Copper Nanoparticles

**Figure 5:**
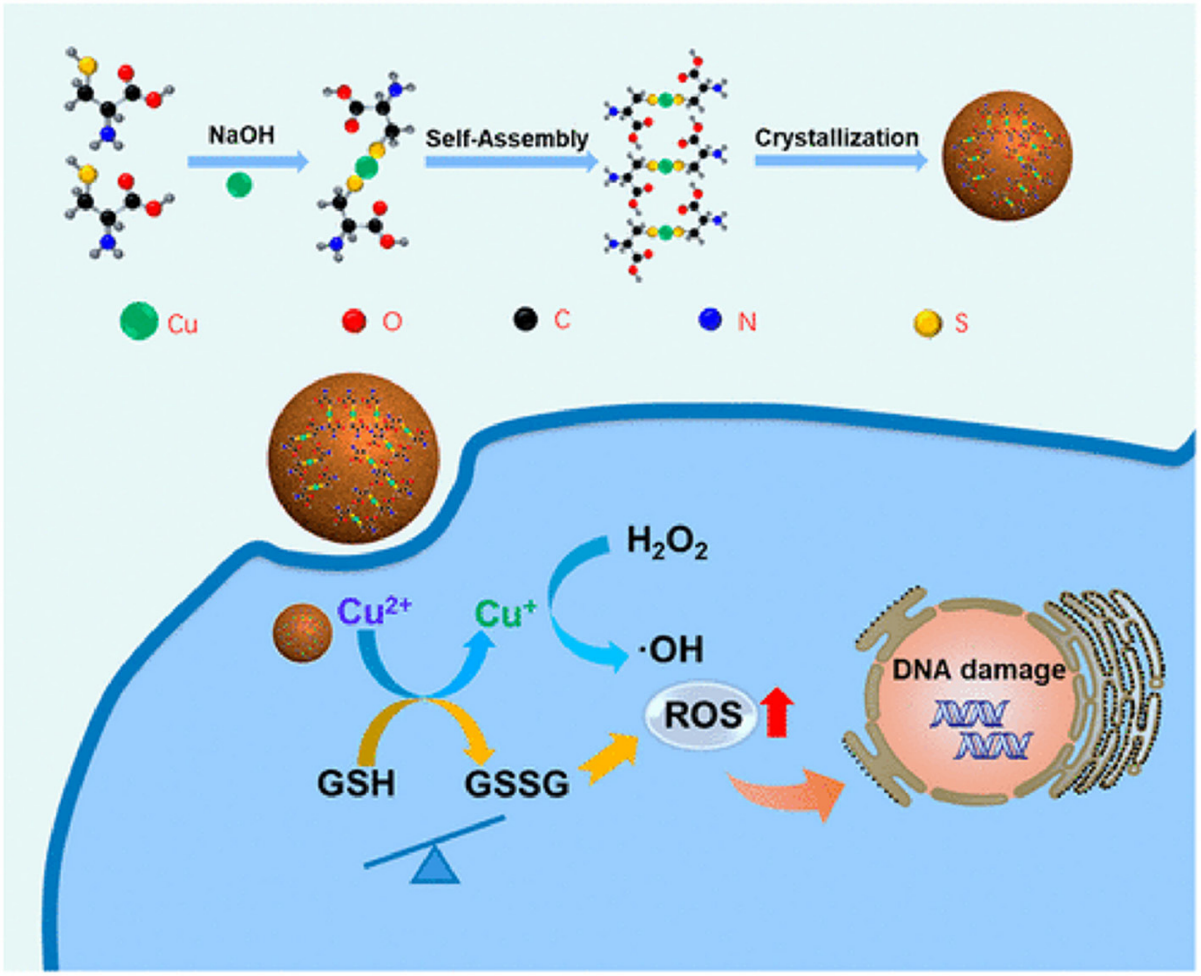
Amino acid-modified copper nanoparticles (CuNPs) induce anticancer effects. CuNPs oxidize glutathione (GSH) to glutathione disulfide (GSSG) and generate Cu^+^ ions, further reacting with endogenous peroxidase to generate hydroxyl radicals to kill cancer cells. Reproduced with permission (Ma et al., 2018) *Copyright* © *2019, American Chemical Society*.

Under acidic conditions, the redox-active catalytic property of copper nanoparticles (CuNPs) induces Fenton-like reactions. The Cu^+^-based Fenton-like reaction demonstrated the highest reaction rate, approximately 160 times higher than that in IONPs (Yang et al., 2020b). Hence, it is a popular H_2_O_2_-responsive Fenton catalyst used in cancer therapy. Unlike Fe-based Fenton reactions, Cu-based Fenton-like reactions are independent of pH, with its catalytic activity achieved in circumneutral pH (6.5–7.5). Both Cu^+^ and Cu^2+^ are highly reactive toward H_2_O_2_, where Cu^+^ is responsible for the generation of hydroxyl radicals (Pham et al., 2013). Moreover, the Cu-based Fenton-like reaction and H_2_O_2_-scavenging property suggested a broad range of possibilities for therapeutic approaches. In the human body, copper is a cofactor for several enzymatic redox reactions, shifting between the two states of Cu^2+^ and Cu^+^, with bioactive copper associated with proteins and their functions (Ma et al., 2018). However, excess free copper ions induce harsh side effects and systemic toxicity. Therefore, researchers have designed CuNPs containing Cu^2+^ ions, which are stable in the physiological environment but reduced to Cu^+^ by tumor-specific reductive stimuli. CuNPs demonstrated a stronger coordination capability with sulfhydryl groups. Hence, Baojin et al. developed cystine-modified CuNPs (Cu-Cys). Cu-Cys nanoparticles can oxidize intracellular GSH to GSSG and simultaneously reduce Cu^2+^ to Cu^+^ ions, then Cu^+^ can react with H_2_O_2_ to generate hydroxyl radicals (shown in the Figure 5; Ma et al., 2018). Thus, amino acid modified CuNPs can be triggered by both intracellular GSH and peroxide stimuli. Owing to the Fenton-like activity, Cu-Cys nanoparticles can scavenge both GSH and H_2_O_2_ and produce adequate hydroxyl radicals, causing lipid peroxidation, DNA damage, and cellular apoptosis (Ma et al., 2018). Amino acid-fabricated Cu-Cys nanoparticles are highly stable in physiological conditions, limiting the unnecessary reduction process, and protecting other organs from systemic toxicity. Similarly, tetrakis (4-carboxyphenyl) porphyrin CuNPs (TCPP-Cu) revealed significant singlet oxygen generation within the TME by scavenging both GSH and H_2_O_2_ (Wang et al., 2019a). In the acidic TME, TCPP-Cu efficiently oxidized H_2_O_2_ to peroxyl radicals and Cu^2+^ ions (Wang et al., 2019a). Furthermore, peroxyl radicals spontaneously generate singlet oxygen via a recombination reaction with Cu^2+^ ions. GSH acts as an antioxidant and high concentrations protect cells from ROS-mediated ferroptosis. TCPP-Cu depletes intracellular GSH, inhibiting ROS consumption, and improving PDT efficacy (Wang et al., 2019a). Cu-Cys nanoparticles oxidize intracellular GSH and generate an adequate amount of ROS to induce ferroptosis (Ma et al., 2018). Finally, CuNPs are popularly considered metallic nanotherapeutics to undergo Fenton reactions with additional advantages and efficacies.

## Application of Stimuli-Triggered Metallic Nanotherapeutics in Cancer Therapies

**Figure 6:**
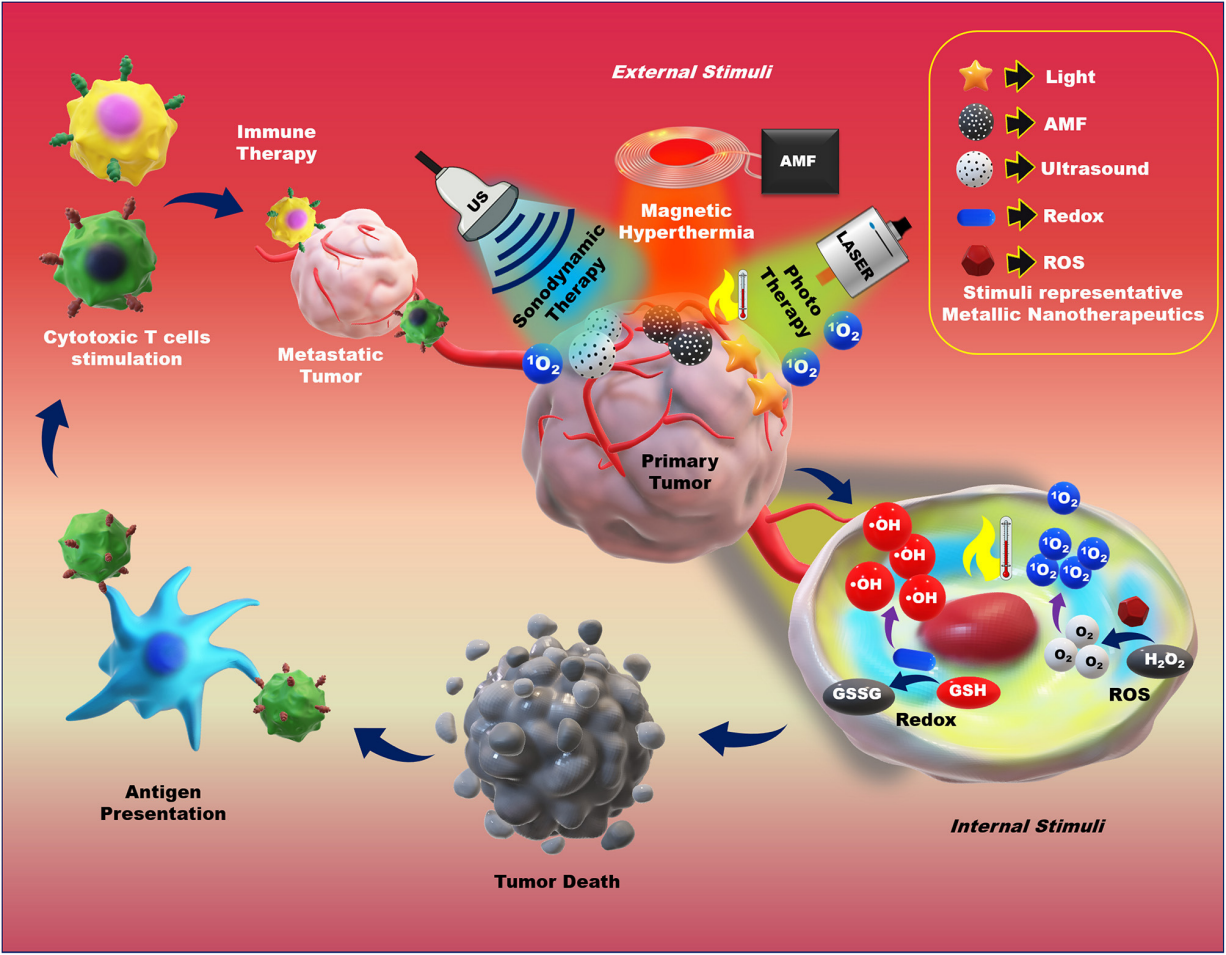
Application of external and internal stimuli-triggered metallic nanotherapeutics for cancer therapy. Different stimuli such as light, an alternative magnetic field (AMF), ultrasound (US), redox, and reactive oxygen species (ROS) trigger metallic nanotherapeutics to exert their anticancer activities. External stimuli such as light, AMF, and US induce phototherapy, magnetic hyperthermia, and sonodynamic therapy, whereas internal stimuli such as redox and ROS promote toxic radical production, resulting in tumor death. Antigens are released upon tumor death and captured by antigen-presenting cells, which further trigger cytotoxic immune cell activation against metastatic tumors.

In cancer therapy, the therapeutic application of MNPs are potentiated by the combination of multiple internal and external stimuli. The controllable anticancer behavior of metallic nanotherapeutics can be achieved by stimuli modulation based on the therapeutic purposes. The surface modification of metallic nanotherapeutics with appropriate moieties can enhance the specificity and reduce the off-site attack against healthy cells, whereas external stimulation can trigger therapeutic activities, resolving the drawbacks of current therapies, and providing a new direction for advanced treatment techniques in cancer therapy (Morales-Cruz et al., 2019; Yoo et al., 2019). Each stimulus affords specific activity, which can be considered during nanomedicine formulation. Here, we discussed the role of metallic nanotherapeutics triggered with external and internal stimuli in multiple anticancer techniques, including PT, CDT, SDT, magnetic hyperthermia, and immune therapy (Figure 6). Some metallic nanotherapeutics have demonstrated responsiveness to multiple stimuli, and diverse anticancer properties, rendering them more applicable in cancer therapy.

### Chemodynamic Therapy

**Figure 7:**
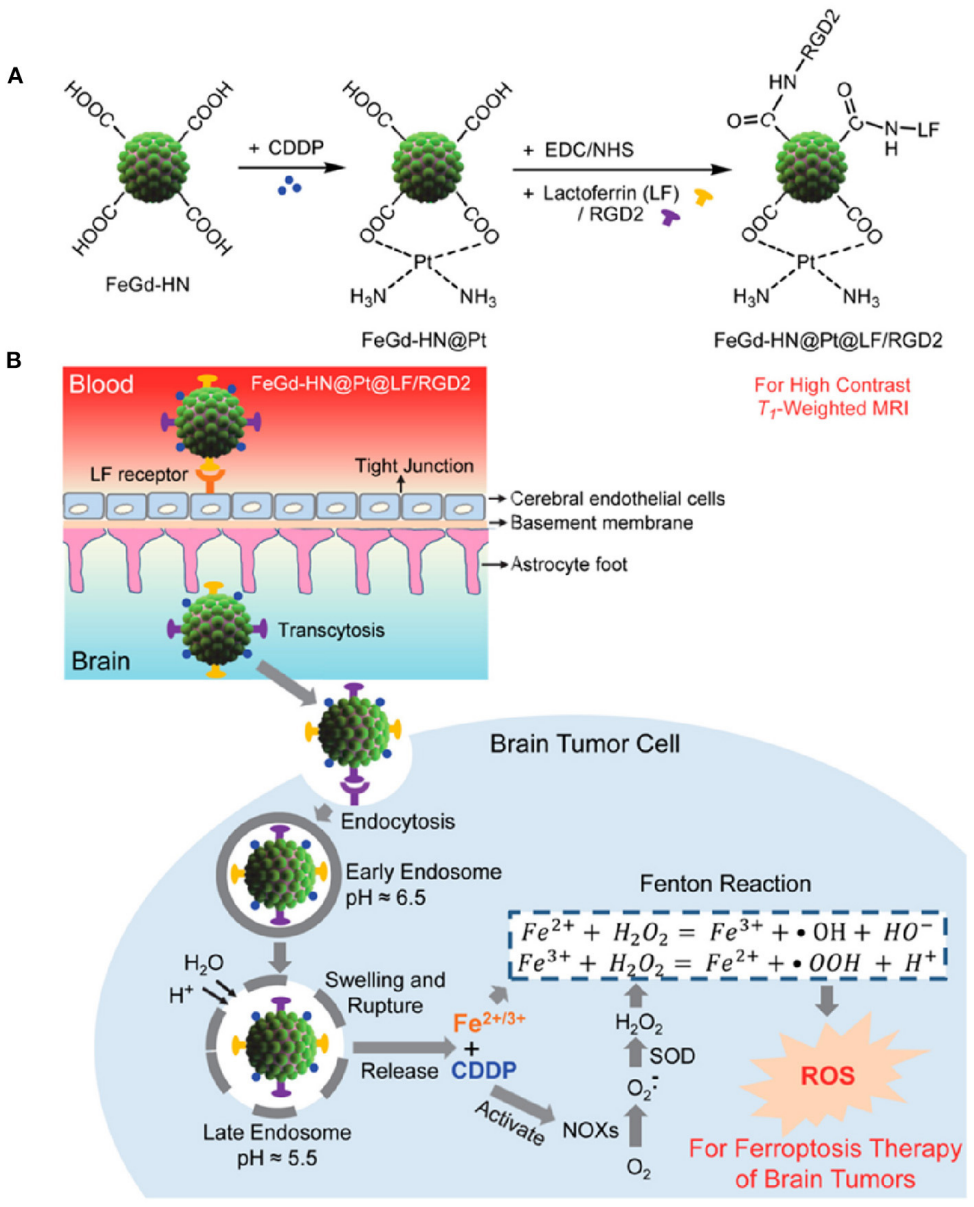
Synthesis procedure of magnetic nanoparticle **(A)**. Mechanism of Brain tumor targeted IONP application in cancer cells via the Fenton reaction **(B)**. Reproduced with permission (Shen et al., 2018). *Copyright* © *2018, American Chemical Society*.

The side effects associated with traditional chemotherapeutic drugs, as well as their efficacy during treatment, cannot be avoided. Therefore, several investigations have focused on nanotechnology-based therapeutic approaches. The TME is highly complex, with hypoxic conditions owing to lower oxygen supply, pH imbalance, and GSH overproduction, as well as the expression of inhibitory proteins, which suppress traditional chemotherapeutic drug efficacy inside the cells, introducing the MDR effect. CDT, an enhanced therapeutic technique involving the generation of hydroxyl radicals (•OH) via the Fenton reaction and Fenton-like reactions, can cause lipid peroxidation, DNA damage, and apoptosis (Liu et al., 2019a). During CDT, metal nanoparticles catalyze H_2_O_2_ to generate hydroxyl radicals (•OH) and molecular oxygen. The main advantages of CDT include GSH depletion and reduced hypoxic conditions in the TME, which are deemed the main source of the MDR effect (Liu et al., 2019a). CDT is based on hydroxyl radical (•OH) generation via an iron oxide-induced Fenton reaction, as well as Fenton-like reactions induced by other metallic nanotherapeutics (CuNPs, MnO2, and GNPs) (Wang et al., 2020c). Hydroxyl radicals are highly reactive, allowing CDT to be extensively investigated for cancer treatment and combinational cancer treatments, enhancing the efficacy of PDT, PTT, and chemotherapy (Hou et al., 2019a). IONPs are the main source, inducing non-apoptotic programmed cell death through iron-dependent ROS generation, i.e., ferroptosis (Dixon et al., 2012). In cancer theranostics, one main research frontier has explored the triggering of *in situ* chemical reactions via endogenous H_2_O_2_ stimuli to produce more toxic hydroxyl radicals via the Fenton reaction (Dixon et al., 2012). SPIONs are ultra-small IONPs widely investigated in cancer therapy and MRI imaging. SPIONs are metabolized in the acidic environment of cancer cells, resulting in the formation of iron ions (Fe^3+^ and Fe^2+^) and undergo endo/lysosomal transportation into the cytosol through divalent metal transporter 1 (Huang et al., 2013). Furthermore, iron ions generate ROS inside the cells via intracellular oxidation-reduction reactions with peroxidase. The combination of SPION-based micelles with the exogenous ROS generator, β-lapachone, catalyzed by overexpressed NAD(P)H:quinone oxidoreductase 1 (NQO1) produced massive amounts of superoxide and H_2_O_2_, elevating antitumor activity by Fenton reaction-based ROS production (Huang et al., 2013). The amount of endogenous H_2_O_2_ is insufficient for the Fenton reaction to generate an adequate amount of ROS to eradicate cancer cells. Shengdi et al. reported that artemisinin (ART) and its derivative, which contains a peroxy group accelerating the Fenton reaction with iron or copper ions, were loaded into MNPs to enhance the cytotoxic effect (Guo et al., 2020a). MNPs are degraded in acidic tumor conditions to release Fe^2+^ ions that induce the Fenton reaction. Under acidic conditions (pH 5.0), the iron content is 10.45 μg/mL, 10 times higher than that in the normal physiological condition of 1.61 μg/mL (Guo et al., 2020a). MNP degradation accelerates the release of ART and its derivatives, generating significant additional peroxidase to enhance the Fenton reaction inside cells. The surface modification of IONPs can achieve specific targeting to brain tumors and cross the blood-brain barrier (BBB) (Shen et al., 2018). Zheyu et al. developed cisplatin (CDDP)-loaded Fe_3_O_4_/Gd_2_O_3_ hybrid nanoparticles with lactoferrin (LF) and RGD dimer surface modifications to treat brain tumors (Shen et al., 2018). The LF functionalization promoted LF receptor-mediated transcytosis through the BBB, with the RGD dimer accelerating cellular endocytosis to transport the FeGd-HN@Pt cargo efficiently. The successful delivery of FeGd-HN@Pt into the brain tumor modulated the release of Fe^3+^, Fe^2+^, and CDDP owing to the acidic environment (shown in Figure 7). Then, CDDP was involved in the NADPH oxidation process, converting NADPH to NADP^+^ by releasing excess superoxide ions and H_2_O_2_, accelerating ferroptosis activity in the brain tumor (Shen et al., 2018).

### Sonodynamic Therapy

For the last two decades, SDT has been established as a non-invasive targeted cancer therapy (Canavese et al., 2018). The principle of SDT is similar to that of PDT and can be used with low-intensity US and a sonosensitizer to generate toxic free radicals (Canavese et al., 2018). As discussed in section Ultrasound (US)-Responsive Nanoparticles, US is a form of mechanical waves that can penetrate deep tissues to activate sonosensitizers (Canavese et al., 2018) and is deemed superior to visible and NIR light-triggered PT because of its enhanced deep tissue penetration property. The SDT mechanism is dependent upon the experimental procedure, biological models, the sonosensitizer type, and the US exposure guidelines including the intensity and frequency used. Various possible principles have been reported for SDT, including free radical generation, ultrasonic cavitation-induced microbubbles, US-induced cell apoptosis, and singlet oxygen production (Mchale et al., 2016). Ultrasonic cavitation is a physical phenomenon induced by immediate pressure changes in the surroundings, resulting in microscopic bubbles in tissues (Mchale et al., 2016). Microscopic tissue bubbles can oscillate, expand, and finally collapse violently in the tissue. Furthermore, ultrasonic cavitation can cause sonoluminescence and generate various sonochemical species such as free radicals and singlet oxygen molecules (Mchale et al., 2016). Ultrasonic sonosensitizer-derived free radicals are generated by reacting with H^+^ and OH^−^ ions produced during the thermolysis of water. These free radicals can react with oxygen and produce peroxyl and alkoxyl radicals, leading to lipid peroxidation and cell death. During SDT, sonosensitizers excite and return to the ground state by releasing energy. The surrounding oxygen molecules accept the released energy and become excited singlet oxygen molecules, which are highly reactive and trigger oxidation reactions inside tumor cells, as well as sufficient singlet oxygen species to initiate various biological activities, including DNA fragmentation, the shrinkage of cytoskeletal filaments, and chromatin condensation to induce cellular apoptosis (Mchale et al., 2016). Reportedly, HIFU can ablate tumors at temperatures up to 80°C, causing thermal toxicity and irreversible cell damage.

**Figure 8:**
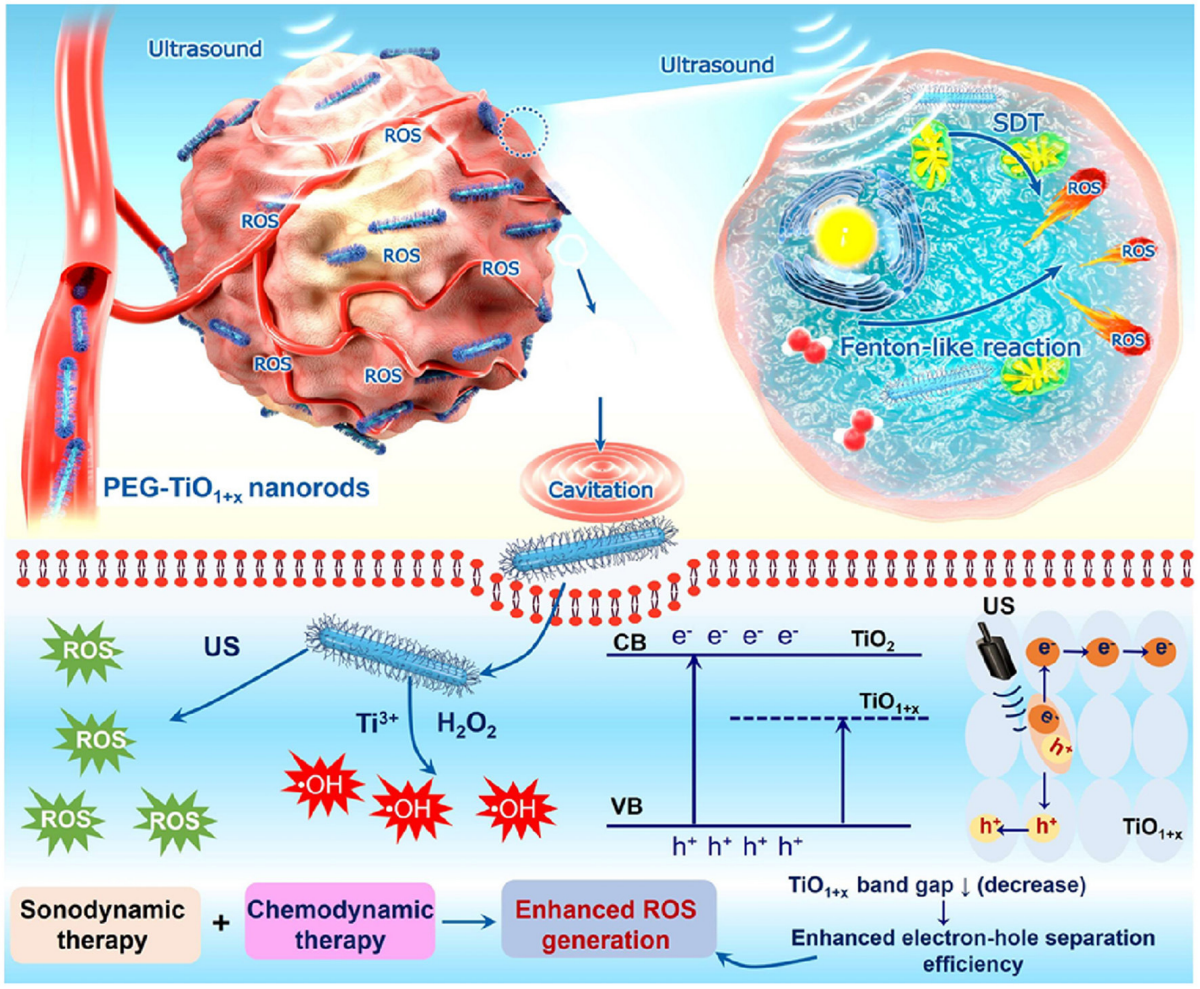
Application of PEGylated titanium monoxide nanorods (TiO_2_ NRs) in sonodynamic therapy. Oxygen-deficient TiO_2_ NRs have shown higher reactive oxygen species (ROS) generation upon ultrasound irradiation. Reproduced with permission (Wang et al., 2020d) *Copyright* © *2020, American Chemical Society*.

Various porphyrin-based organic sonosensitizers are utilized for SDT against cancer. However, organic sonosensitizers are quickly degraded by ROS. Hence, TiO_2_ nanoparticles are considered biocompatible MNPs as they are chemically inert and highly stable in the physiological environment (You et al., 2016). As a potential alternative to PDT, carboxymethyl dextran (CMD)-modified hydrophilic TiO_2_ (HTiO_2_) has been investigated for US-triggered SDT (You et al., 2016). CMD modification provides a unique stealth characteristic to TiO_2_ nanoparticles, enhancing the blood circulation time (You et al., 2016). CMD-coated HTiO_2_ demonstrated a hydrodynamic size of 198 nm, with a negative surface charge and good stability in the physiological environment (pH 7.4) for 5 days (You et al., 2016). HTiO_2_ nanoparticles caused significant *in vitro* and *in vivo* ROS generation under US exposure in a dose-dependent manner, elevating the ROS level sufficiently to destroy tumor blood vessels and suppress tumor growth. Furthermore, HTiO_2_ nanoparticle-based SDT upregulated the levels of pro-inflammatory cytokines such as interleukin (IL)-1β, IL-6, and tumor necrosis factor (TNF)-α within the tumor, increasing the immune response against the tumor. Additionally, the hollow mesoporous structure possesses an additional function, i.e., carrying therapeutic cargoes inside for combinational drug delivery and SDT (Feng et al., 2019). During cancer treatment, SDT is involved in both apoptosis and autophagy mechanisms. During SDT, autophagy acts as a double-edged sword, suppressing tumor growth in the early stage, but inducing tumor cell progression and suppressing SDT-mediated apoptosis in the later stage (Feng et al., 2019). Feng et al. developed hollow mesoporous titanium dioxide nanoparticles (HMTNPs) loaded with hydroxychloroquine (HCQ), an autophagy inhibitor, for autophagy-suppressed SDT (Feng et al., 2019). Furthermore, HMTNPs were coated with cancer cell membrane (CCM) to induce biomimetic behavior, which can safeguard against macrophage phagocytes and tumor homing activities. In the tumor, ultrasonic stimuli triggered HCQ release and suppressed autophagy by blocking the damaged tissue-derived nutrient supply to cancer cells, promoting SDT resistance (Feng et al., 2019). Subsequently, CCM-HMTNPs/HCQ demonstrated strong ROS generation and SDT sensitivity against a breast cancer model with US triggering. However, TiO_2_ nanoparticles exhibit a low quantum yield for ROS generation. Hence, hybrid titanium-based sonosensitizers have been investigated for cancer SDT (Feng et al., 2019). Wang et al. reported nanorod-shaped titanium monoxide nanoparticles (TiO_1+x_), which exhibited potent US-triggered ROS generation owing to an oxygen-deficient arrangement (Wang et al., 2020d). An oxygen-deficient structure provides a charge trap to reduce the band gap and limit electron-hole recombination, resulting in elevated ROS generation during US exposure (Wang et al., 2020d). PEGylated TiO_1+x_ nanorods (PEG- TiO_1+x_ NRs) demonstrated a horseradish peroxidase-like nanozyme activity, generating hydroxyl radicals (•OH) by reacting with H_2_O_2_ via a Fenton-like reaction. Reportedly, the intravenous administration of PEG-TiO_1+x_ NRs to a 4T1 tumor mouse model enhanced ROS generation under US irradiation (40 kHz, 3.0 W/cm^2^) for 5 min. ROS generation via SDT and hydroxyl radical (•OH) generation by the Fenton-like reaction (Figure 8) successfully inhibited tumor growth, which was superior to TiO_2_-based nanoparticle treatment (Wang et al., 2020d). Mitochondria are considered the powerhouse of cells and produce ROS to regulate cellular metabolism. An imbalance in ROS in the mitochondria leads to mitochondrial dysfunction and cancer cell apoptosis. Cao et al. reported that mitochondrial-targeted TiO_2_ nanosheets efficiently inhibited tumor growth (Cao et al., 2019). TiO_2_ nanosheets consist of highly reactive facets, decorated with Au crystals, and modified with a cancer cell-targeting aptamer (AS_1411_) and triphenylphosphine (TPP) to target mitochondria inside the cells. These TiO_2_ nanosheets induced Au crystal growth, limiting the fast recombination of excited electrons and holes (Cao et al., 2019). Furthermore, Au crystals present on the surface-mediated interfacial electron transfer to promote a high ROS quantum yield under US exposure. Dual-targeting Au-TiO_2_-A-TPP nanosheets have demonstrated complete tumor regression without tumor relapse (Cao et al., 2019).

### Magnetic Hyperthermia

Magnetic nanoparticle-induced hyperthermia is aimed at raising the tumor tissue temperature up to 40–43°C, inducing protein and DNA impairment, and resulting in cancer cell death (Chang et al., 2018). MNPs convert magnetic energy to thermal energy upon AMF exposure owing to the loss of heat during the reversal of the magnetization process. Three major mechanisms are involved, hysteresis loss, eddy current, and Néel and Brownian relaxation mechanisms, which conduct the heat loss process and thermal energy production following AMF exposure (Hervault and Thanh, 2014). Magnetic heating based on hysteresis loss is developed using multi-domain MNPs, i.e., ferromagnetic or ferrimagnetic nanoparticles, 100 nm in size. The ferromagnetic material is incorporated with a uniform magnetic domain aligned in one direction, whereas ferrimagnetic material contains magnetic domains aligned opposite to each other. Following the application of an external magnetic field, the magnetic domain magnetizes in the same direction during the positive half-cycle, demagnetizing during the negative half-cycle. Withdrawal of the applied magnetic fields does not revert the magnetization to zero, for which the external intensity of the magnetic field is required. The sequence of the magnetization presents as a non-linear curve, which is termed the hysteresis loop, with the curve representing the magnetic strength of the MNPs (Hervault and Thanh, 2014). Superparamagnetic MNPs are small (10–20 nm) and contain only a single domain. During withdrawal of the magnetic field, superparamagnetic MNPs do not retain any magnetization and dissipate heat through relaxation loss based on Néel and Brownian relaxations (Hervault and Thanh, 2014). Depending upon the temperature and duration of hyperthermia, AMF-induced hyperthermia can cause cancer cell death. Hyperthermia is involved in various changes observed within cells such as protein denaturation and aggregation, as well as the regulation of several downstream pathways involved in the synthesis of cellular proteins, cell growth, and DNA repair. Elevated temperatures induce blood vessel perfusion, resulting in increased blood circulation, as well as the delivery of oxygen and chemotherapeutic moieties (Chang et al., 2018). Similarly, AMF-induced magnetic hyperthermia provokes an immune response inside tumors through cytotoxic immune cell infiltration, as well as pro-inflammatory cytokine production within cancer cells (Chang et al., 2018). Folic acid-modified PEGylated SPIONs (Mag-PEG-FA) are suitable candidates to target cancer cells overexpressing folic acid receptors (Piazza et al., 2020). Mag-PEG-FA NPs, with a hydrodynamic size of 94 nm, are highly stable in various physiological conditions (Piazza et al., 2020), demonstrating the highest surface absorption rate (SAR) of 21.6 W g^−1^ following an increase in temperature to 42°C upon 80 s of AMF exposure (Piazza et al., 2020). To achieve combined magnetic hyperthermia and chemotherapy, Sivakumar et al. used SPIONs and curcumin, an anticancer drug, encapsulated within poly(lactic-co-glycolic acid) (PLGA) nanoparticles with a surface modification using the pancreatic cancer-specific AS1411 aptamer (Sivakumar et al., 2017). SPION nanocomposites are excellent material for T2 contrast-based MRI and PA imaging, introducing a theranostic approach. Following the induction of hyperthermia, pancreatic cancer cell-targeted nanoparticles effectively unloaded the anticancer drug within the cells (Sivakumar et al., 2017). MNPs are considered an efficient drug delivery agent for site-specific on-demand delivery. Kondareddy et al. developed magnetic field-inducible drug-eluting nanoparticles (MIDENs) for the delivery of DOX dual cancer therapy. DOX and SPIONs were loaded into a temperature-responsive PLGA nanoparticle (T_g_ = 42–45°C) (Thirunavukkarasu et al., 2018). Following AMF exposure, the MIDENs elevated the temperature above 45°C, which facilitated the transition of the PLGA polymer matrix from a glassy to a rubbery state, resulting in the release of DOX inside colon cancer cells (Thirunavukkarasu et al., 2018). A significant release of DOX delivered by MIDENs can be promoted upon AMF stimulation and control non-specific toxicity within the body. AMF-induced combinational magnetic hyperthermia and chemotherapy is a potential anticancer strategy to eradicate tumors and enable *in vivo* tracking by MRI (Thirunavukkarasu et al., 2018).

**Figure 9:**
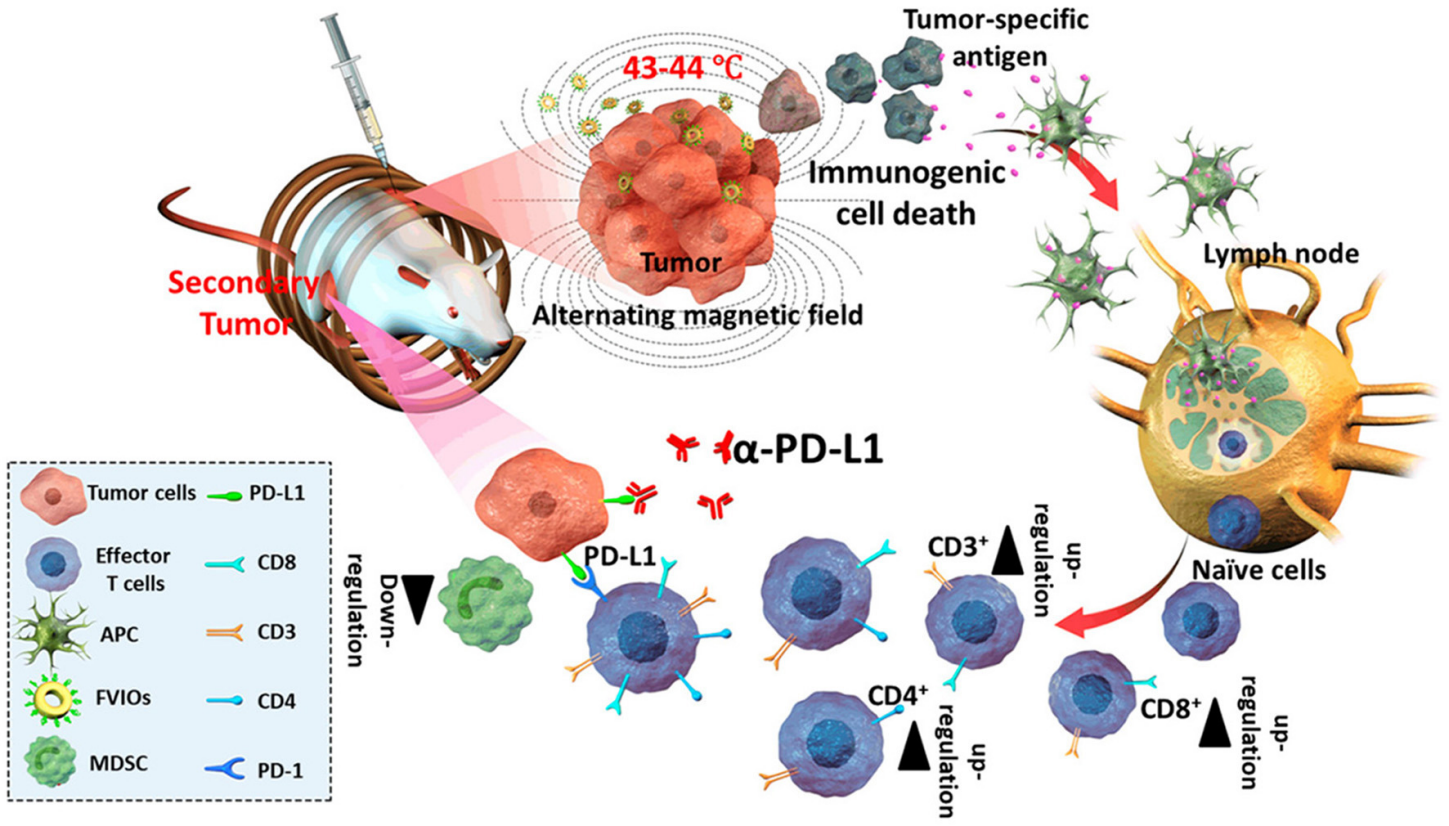
Alternative magnetic field (AMF)-induced hyperthermia and immune therapy. AMF-induced heat triggers immunogenic cell death (ICD) and antigen release in tumors, which further induces cytotoxic T lymphocyte activation, and the combination with checkpoint blockade activates antitumor immune therapy. Reproduced from Liu et al. (2019b) *Copyright* © *2019, American Chemical Society*.

Superparamagnetic MNPs possess properties leading to aggregation, with weaker magnetization hysteresis loops suitable for biomedical applications, but lower magnetic power than larger MNPs. However, larger-sized MNPs can provide higher magnetic power but tend to aggregate following AMF exposure (Pardo et al., 2020). Hence, modifying the magnetic property of MNPs by tailoring their morphology and composition is an important aspect that needs consideration. Recently, hybrid ferrite nanoparticles were developed by substitution with other magnetic atoms, including cobalt (Co), manganese (Mn), nickel (Ni), gadolinium (Gd), and yttrium (Yt), onto the tetrahedral A and octahedral B sites of the spinal structure of magnetite, modulating the magnetic behavior and thermal ablation properties (Pardo et al., 2020). Elvira et al. reported that the controlled doping of Co(II) into ferrite nanoparticles significantly enhanced the magnetic hyperthermia efficiency (Fantechi et al., 2014). Human ferritin (HFt) is a protein involved in iron homeostasis in the body and can be safely assembled into a cage-like structure, with a 12-nm outer layer and an 8-nm inner cavity. HFt can be easily functionalized with diverse moieties and acts as a favorable template for the biomineralization of various MNPs (Fantechi et al., 2014). A small amount of Co (5%) doping in HFt-based ferrite nanoparticles reportedly enhanced the SAR value and hyperthermia effect (Fantechi et al., 2014). Furthermore, the therapeutic performance of co-doped ferrite nanoparticles has been investigated following AMF exposure under physiological tolerance for 30 min, demonstrating significant cytotoxic behavior compared to undoped HFt nanoparticles in melanoma cancer cells (Fantechi et al., 2014). Hasan et al. reported that Co and Mn-doped hexagonal structured IONPs demonstrated 3.6-times higher SAR values than those of spherical IONPs (CoMn-IONP, 1718.0 W/g; IONP, 475.3 W/g) (Albarqi et al., 2019). For intravenous administration, CoMn-IONPs are coated with a poly(ethylene glycol)-b poly(ε-caprolactone) (PEG–PCL)-based polymer for undisturbed tumor targeting and biocompatibility. Compared to commercially available IONPs, CoMn-IONPs demonstrated high heating efficiency under AMF exposure (frequency, 420 kHz; magnetic field strength, 26.9 kA/m). In this process, the temperature of the CoMn-IONPs increased to 40°C within 10 min, whereas the temperature of spherical IONPs only increased to 20°C (Albarqi et al., 2019). Additionally, the anticancer effect of CoMn-IONPs was evaluated in ovarian cancer cells with a non-toxic concentration of 50 μg/mL for 24 h. Under AMF exposure, CoMn-IONPs increased the temperature up to 46°C, inducing 99% cell toxicity, whereas spherical IONPs caused a 10% reduction in cell viability (Albarqi et al., 2019). Similarly, shape modulations can regulate the SAR value of MNPs and effectively contribute to magnetic hyperthermia. Liu et al. developed ferrimagnetic vortex-domain iron oxide nanorings (FVIOs) that attained a markedly high SAR value (3,000 W/g), twelve times higher than that of the FDA-approved IONPs (Liu et al., 2019b). Under AMF exposure, PEGylated FVIOs have demonstrated a significant cell death of 38.42% during early apoptosis and 48.96% in late apoptosis. Furthermore, mild hyperthermia induces calreticulin (CRT) expression, transmitting an “eat-me” signal to immune cells, further activating ICD, and resulting in effective immune therapy (Figure 9; Liu et al., 2019b). AMF-treated FVIOs demonstrated significantly higher CRT expression than that of the non-treated group, as well as the production of multiple pro-inflammatory cytokines. Furthermore, ICD-induced immune reactions revealed enhanced cytotoxic T lymphocyte (CTL) infiltration, including CD8+ and CD4+ cells, into the tumor site. AMF treatment combined with programmed death-1 (PD-1) ligand 1 (PD-L1) checkpoint blockade eradicated both primary and secondary tumors in the abscopal 4T1 model, as well as lung metastasis in a metastatic model (Liu et al., 2019b).

### Phototherapy

**Figure 10:**
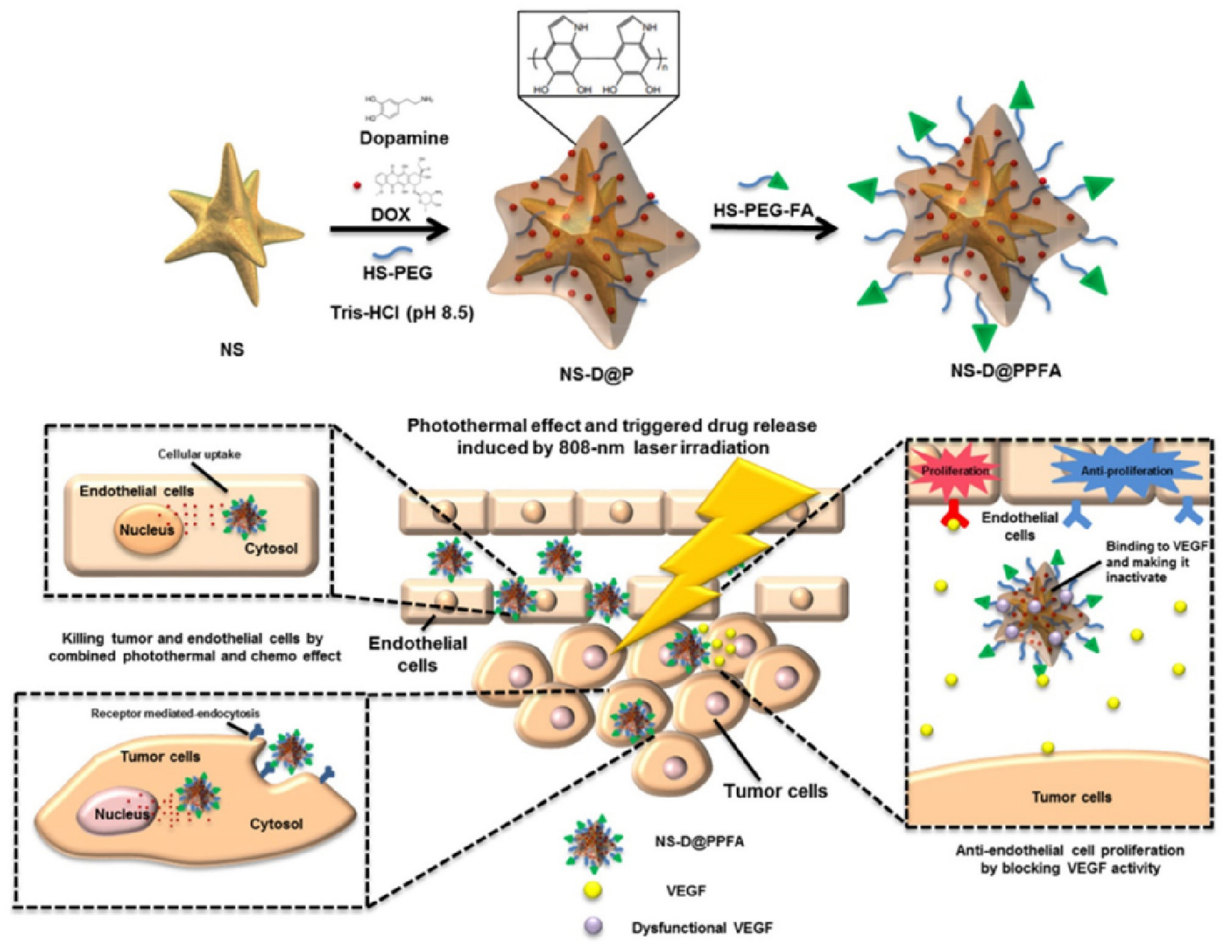
Gold nanostars (GNS) modified with folic acid and thiolated polyethylene glycol (PEG) for targeted cancer therapy. GNS coated with dopamine can facilitate doxycycline (DOX)-loading and are modified with targeting ligands for near-infrared (NIR)-triggered drug release and photothermal chemotherapy. Reproduced with permission from You et al. (2019) under *copyright Creative Commons Attribution 4.0 International License (CC-BY license)*.

PTT is a minimally invasive and prominent therapeutic approach, involving a photosensitizer that can absorb light energy and generate heat and ROS, which is triggered using electromagnetic radiation such as NIR or visible light. In the cellular environment, heat causes hyperthermia, resulting in protein denaturation and protein aggregation, cell lysis, and cytosol evaporation, which leads to cancer cell death. PTT activates programmed cell death following a specific temperature rise within the cell but avoids necrotic cell death, hindering antitumor activities. Similar to PTT, PDT relies on the generation of various ROS, including hydroxyl radicals, singlet oxygen, and superoxide ions. PDT-induced ROS interacts with various cellular chemical reactions and pathways, resulting in cancer cell death. Photosensitizers like organic dyes, semiconducting polymers, and MNPs have been investigated in phototherapy. Among the available photosensitizers, MNPs are potent agents as they can be stimulated multiple times to generate heat and ROS, whereas photosensitizing dyes are degraded by single irradiation. GNPs are widely used for cancer theranostic applications and as anti-angiogenic agents. GNPs interfere with angiogenesis-based signaling pathways through vascular endothelial growth factor (VEGF) binding with the sulfur or amine groups of amino acids present in the heparin-binding domain, thus initiating the inhibition of angiogenesis in the tumor tissue (You et al., 2019). Huang et al. reported that GNS, featuring multiple sharp tips, induced tip-enhanced plasmonic effects and high photothermal conversion efficiency in PTT (You et al., 2019). Furthermore, the polydopamine coating of GNS enabled DOX loading through electrostatic or π-π stacking interactions, accelerating photothermal efficiency (Figure 10). Surface modification with folic acid-tethered thiol polyethylene glycol (HS-PEG-FA) resulted in specific targeting to MCF-7 breast cancer cells for dual chemo-PTT. In response to NIR (808 nm, 0.9 W/cm^2^) laser irradiation, GNS generated a strong PTT effect and triggered drug release. Combined chemo-PTT showed marked efficacy in MCF cells, a breast cancer model, and the drug-resistant MCF-7/ADR tumor model (You et al., 2019). Furthermore, folic acid-modified GNS successfully inhibited VEGF-mediated angiogenesis and reduced the levels of CD31 and pVEGFR2 in tumor xenograft tissues (You et al., 2019). Lee et al. demonstrated that gold nanoshells were efficient drug carriers and strongly absorbed in the NIR region (Lee and Shieh, 2020). In colorectal cancer, platinum (II) drug-loaded gold nanoshells ablated the tumor region under NIR laser exposure at a mild power density (1W/cm^2^), triggering drug release in chemo-PTT combinational treatment (Lee and Shieh, 2020). Similarly, Weijun et al. developed NIR/pH dual-responsive gold nanorod-based nanotherapeutics for combined chemo-PTT in a breast cancer model (Xu et al., 2019b). GNRs have been modified with dopamine-functionalized hyaluronic acid via Au-catechol binding. Additionally, DOX was anchored on hyaluronic acid and modified with hydroxyethyl chitosan, a pH-dependent cationic polysaccharide that modulated the GNR-HA^DOX^CH-based nanotherapeutic surface charge from negative to positive in the acidic tumor environment. Under NIR laser (2.0 W/cm^2^) irradiation for 10 min, GNR-HA^DOX^CH elevated the temperature up to 55°C and triggered a 60% drug release, which was 1.8-fold higher than that in the non-irradiated group (Xu et al., 2019b). Dual pH/NIR-responsive GNR-HA^DOX^CH revealed a higher cellular accumulation owing to CD44-based targeting and surface charge reversal, demonstrating a significant cytotoxic effect.

Other than GNPs, copper sulfide-based nanoparticles are gaining significant attention owing to their combinational PTT and PDT efficacies. Wang et al. demonstrated that copper-deficient copper sulfide nanoparticles (Cu_2−x_S) combined with different metal domains were superior photothermal conversion agents owing to their SPR property (Wang et al., 2015b). NIR-triggered Cu_2−x_S could convert light to heat energy, simultaneously generating ROS (Wang et al., 2015b). PEGylated Cu_2−x_S nanocrystals were intratumorally injected into B16 tumor-bearing mice to measure the PTT effect. Following irradiation with an NIR laser for 100 s, Cu_2−x_S nanocrystals elevated the tumor temperature to 47.1°C, which was sufficient to kill tumor cells (Wang et al., 2015b). The photothermal efficiency was evaluated through heat shock protein 70 (Hsp70) expression, as it can be activated by temperature and environmental stress. The laser-irradiated Cu_2−x_S nanocrystal group demonstrated significantly higher Hsp70 expression than that in the non-irradiated group. Moreover, dichlorofluorescein (DCFDA) and electron-spin resonance (ESR) spectral analysis confirmed ROS generation under laser irradiation. Hence, the combined PDT and PTT efficiency of copper MNPs can be further investigated for cancer therapy (Wang et al., 2015b). Alberto et al. developed iron oxide nanoflower-based copper shell-like nanoparticles (IONF@CuS) for PA and MRI imaging and combined PTT, PDT, and magnetic hyperthermia (Curcio et al., 2019). IONFs with copper shells demonstrated photothermal efficiency, acting as a bimodal nano-heater under AMF and NIR irradiation. Tri-therapeutic strategies have successfully eliminated tumors both *in vitro* and *in vivo* (Curcio et al., 2019). Similarly, other MNPs, including Mo, Pd, Pt, Mn, and Bi nanoparticles, have been extensively investigated as excellent phototherapy and imaging agents, which can be used in clinical studies based on their biocompatible behaviors (Badrigilan et al., 2020; Guo et al., 2020b; Jiang et al., 2020; Qian et al., 2020).

### Combinational Immunotherapy

The mechanism of cancer immunotherapy is based on the therapeutic activity of the body's immune system against cancer to eradicate tumor cells (Yang et al., 2020a). During cancer cell death, the released tumor antigens are captured by antigen-presenting cells (APCs) such as immature dendritic cells (iDC) and infiltrate tumor-draining lymph nodes (Park et al., 2018). In the lymph nodes, the antigens are exposed to T cells through major histocompatibility complex (MHC) I or II through mature DCs. APCs activate naive T cells via MHC binding to T cell receptors and costimulatory molecules like CD80 and CD86 on APCs bind to the CD28 receptor on T cells, releasing cytokines that stimulate T cells. Activated T cells are divided into two subpopulations, CD4+ T cells and CD8+ T cells. CD4+ T cells differentiate into T helper cells (Th I or Th II) and stimulate natural killer cells (NK cells) and macrophages that eliminate tumor cells and release pro-inflammatory cytokines to initiate the inflammatory process. CD8+ T cells are divided into CTLs and memory cells to induce potent anticancer immunity. However, various obstacles limit immune activity against cancer. During cancer cell elimination, dead cancer cells release immune suppressive factors such as IL-10, transforming growth factor (TGF)-β, and sphingosine-1-phosphate (S1P), which reduce the M1 macrophage population inside the tumor by polarizing M1 cells to M2 cells (Park et al., 2018). Apoptotic cancer cells recruit monocytes inside the tumor by secreting chemo attractants, which further differentiate into TAMs. The infiltration of MDSCs and TAMs into the tumor simultaneously suppresses the immune response. Furthermore, T cell activity is hindered owing to the expression of tumor-suppressive molecules such as PD-L1 and PD-L2 on cancer cells and CTLA-4 and PD-1 on T cells, which camouflage tumor cells from the immune system, and ultimately limit anticancer immunity. To overcome this barrier, scientists have developed nano-sized vehicles to deliver antigens and adjuvants successfully, which remodel the TME and induce cytotoxic activities and a pro-inflammatory process (Park et al., 2018). MNPs are perceptive agents that can be modified with various ligands to induce specific targeting and delivery (Evans et al., 2018). Stimuli-responsive MNPs can achieve successful antigen and adjuvant delivery, performing diverse therapeutic activities to stimulate antigen release (Evans et al., 2018).

**Figure 11:**
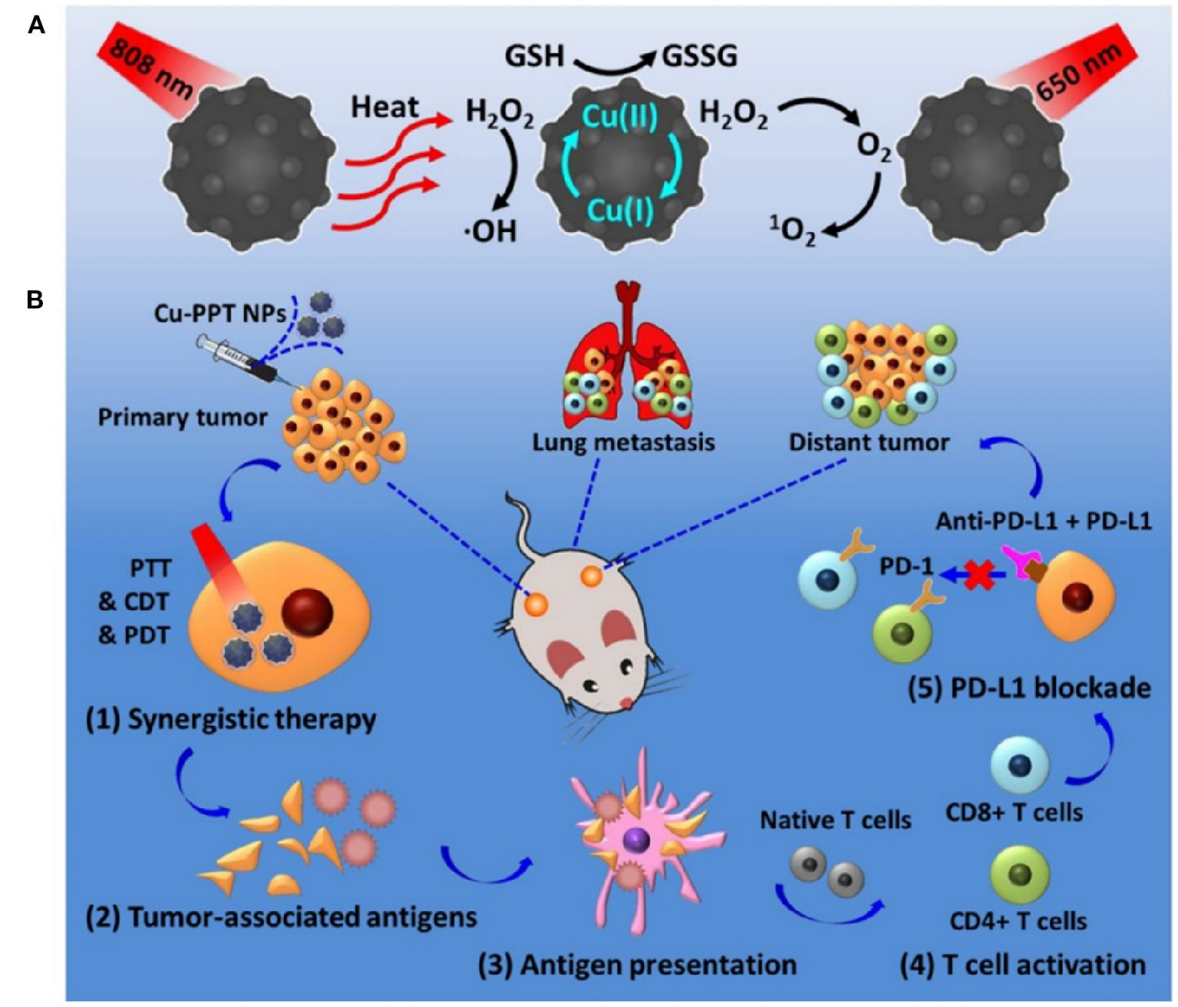
Schematic representation of Cu-PPT based synergistic anticancer effect with photothermal therapy, photodynamic therapy, and chemodynamic therapy **(A)**. Mechanism of Synergistic therapy triggers the release of tumor-associated antigens and PD-1/PD-L1 combination induced a robust antitumor immunity **(B)**. Reproduced with permission from Hu et al. (2020) under *Copyright* © *2020, American Chemical Society*.

**Figure 12:**
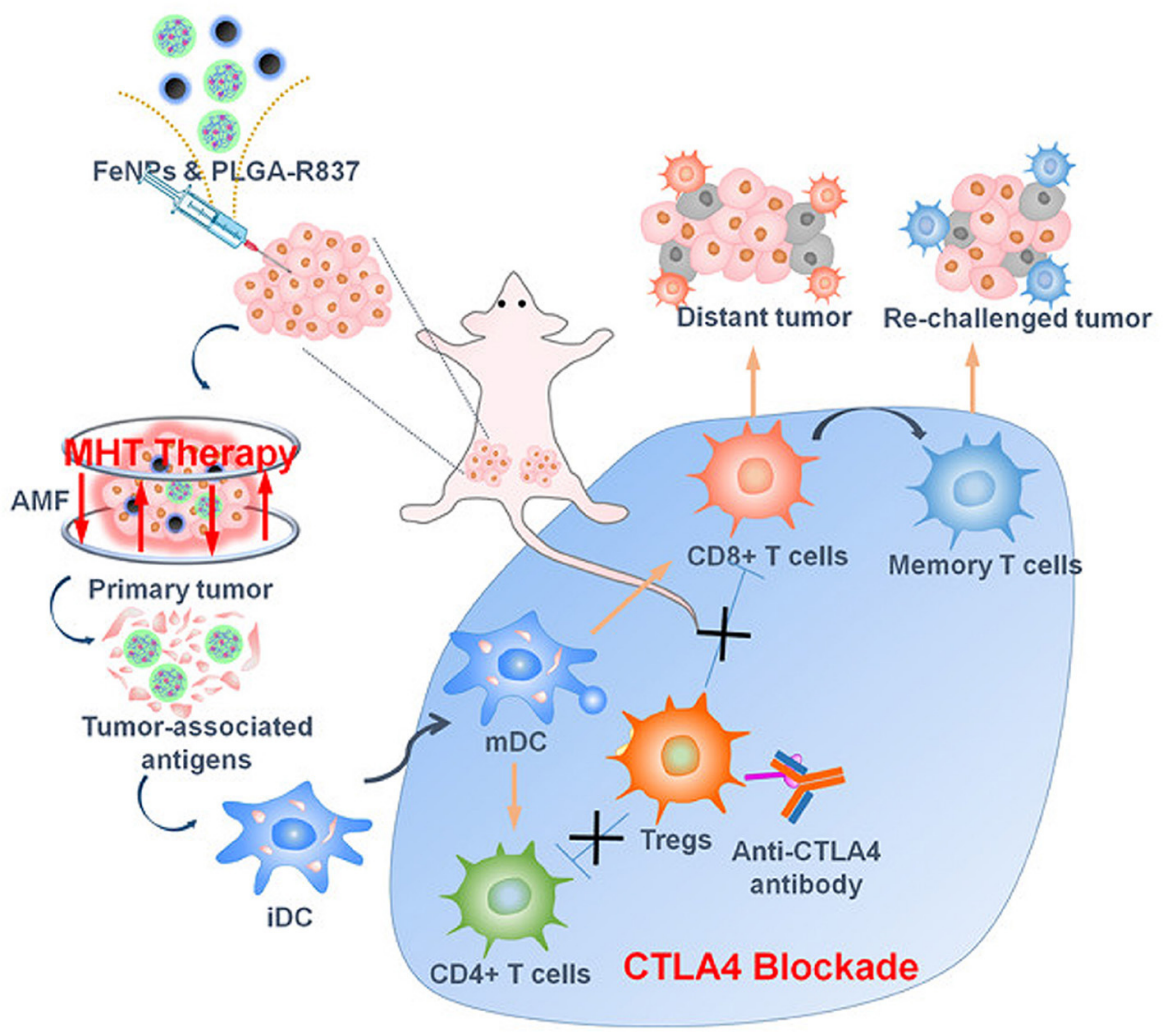
Mechanism of toll-like receptor (TLR)-7 agonist-based iron oxide nanoparticle (IONP) delivery with a checkpoint blockade antibody for alternative magnetic field (AMF)-induced magnetic hyperthermia and immune therapy. Reproduced from Chao et al. (2019) *Copyright* © *2019, American Chemical Society*.

Stimuli-triggered metallic nanotherapeutics like CDT, SDT, PTT/PDT, and RT provide cancer therapy in association with immune therapy directly or indirectly by promoting ICD, which releases antigens, or in combination with various immune stimulatory molecules and immune-suppressive inhibitors (Park et al., 2018). Kang et al. reported size-dependent GNP-based vaccine delivery to lymph nodes, inducing immune response activity (Kang et al., 2017). Ovalbumin (OVA), a primary antigen, conjugated to GNPs (OVA-GNPs) of different sizes such as 10 nm, 22 nm, and 33 nm, demonstrated size-dependent DC uptake and T cell presentation (Kang et al., 2017). OVA-GNPs with sizes of 22 nm and 33 nm demonstrated higher antigen delivery to lymph nodes than 10 nm-sized OVA-GN. Furthermore, 22 nm-sized OVA-GNPs induced a stringent antitumor response and CD8+ T cell infiltration into tumors (Kang et al., 2017). Nanoparticle-based PTT ablated the tumor, and induced ICD and tumor-associated antigens (TAA) to remodel cold tumors into hot tumors and promote T cell infiltration. Wang et al. developed gold nanostar (AuNSs)-encapsulated selenium nanoparticles (Au@Se nanoparticles) via gold-selenium coordination for PTT-induced immunotherapy (Wang et al., 2020a). Selenium coordination on the AuNSs enhanced the photothermal conversion effect through AuNSs plasmonic coupling, and NIR induced heat-activated SeNP activities, promoting cellular secondary metabolism and apoptosis (Wang et al., 2020a). During NIR irradiation, the Au@Se nanoparticles upregulated HSP 70 and TAA expression to induce CTL infiltration by antigen cross presentation (Wang et al., 2020a). Furthermore, Au@Se nanoparticles reprogrammed TAMs from M2 to M1 types, accelerating pro-inflammatory activities inside the tumors, and re-challenged distant tumor growth. Au@Se nanoparticle-induced PTT could introduce an efficient strategy to remodel the immunosuppressive TME to an immune-supportive TME through infiltrating CTLs and pro-inflammatory cytokine secretion such as IL-12p40 and TNF-α (Wang et al., 2020a). Inhibiting the activities of immunosuppressive Tregs through checkpoint blockade therapy can reinforce the CTL performance against cancer cells and suppress metastatic behavior. However, the lack of deep tissue penetration and nanoparticle accumulation limits PTT efficacy, generating ineffective PTT that further upregulates PD-1 or HSP proteins, and reduces the antitumor immune response (Yang et al., 2019b). Hence, the active targeting of nanoparticles to cancer cells can potentiate the PTT effect, and the combination with checkpoint blockade therapy (PD-1/PD-L1) can suppress the immunosuppressive activity of immune cells inside the TME. Hu et al. presented copper-doped covalent organic polymerized-*p*-phenylenediamine-5,10,15,20-tetra-(4-aminophenyl)porphyrin nanoparticles (Cu-PPT) for enhanced photo-chemodynamic therapy (Hu et al., 2020). Copper-doping induces catalytic activity, including peroxidase-mimicking and Fenton-like activity, to stimulate the CDT effect. Irradiation with both 650 nm and 808 nm laser achieved synergistic PTT and PDT activity. Combinational therapy augmented tumor-associated antigen release and antitumor immunity stimulation (Figure 11; Hu et al., 2020). Moreover, PD-1/PD-L1 checkpoint blockade therapy restricted immune tolerance and T cell stimulation against tumors (Hu et al., 2020). Yang et al. developed matrix metalloproteinase-2 (MMP2)-responsive peptide-modified Au@Pt NPs for tumor homing photo-immunotherapy (Yang et al., 2019b). A tumor-targeting linear peptide sequence (LyP-1) was designed with a PD-L1 antagonist (^D^PPA-1) through an MMP-2 switch, which could trigger PD-L1 antagonist peptide release inside the tumor and expose the LyP-1 peptide on the nanoparticle surface, targeting P32 overexpressed protein on the tumor cell surface. MMP-responsive cleavage of the ^D^PPA-1 sequence allowed binding to the PD-L1 receptor on tumors and prevented PD-1/PD-L1 checkpoint activation, which reduced T cell antitumor activity (Yang et al., 2019b). The PTT treated group revealed significant PD-L1 and MMP expression. Hence, the MMP-responsive ^D^PPA-1 and LyP-1 peptide-modified Au@Pt NP (Au@Pt-LM^D^P)-treated group showed significant tumor inhibition with 100% survival after treatment (Yang et al., 2019b). The Au@Pt-LC^D^P-treated (MMP-responsive switch absent) group showed uncontrolled tumor growth of both the primary and distant tumors after 2 weeks of PTT treatment, attributed to the unsuccessful release of the PD-L1 antagonist (^D^PPA-1), justifying the efficacy of combinational PD-L1 peptide-based immunotherapy and PTT (Yang et al., 2019b). In the previous section, we explained that mild hyperthermia induced by an AMF could trigger ICD and an antitumor response. Upon AMF exposure, the intratumorally delivery of nano-adjuvants and IONPs modulated the immune response and DC maturation (Chao et al., 2019). The delivery of R837 (TLR 7 agonist) stimulated antigen presentation and CTL infiltration inside the tumor (Chao et al., 2019). Combined therapy with AMF exposure triggered mild hyperthermia, and the adjuvant-based treatment successfully initiated a strong immune response against cancer (Figure 12). Reportedly, the systemic administration of a checkpoint antibody (anti-CTLA4) inhibited the immune suppressive molecules that heavily influence the immune response against cancer cells (Chao et al., 2019).

A combination of antigen and adjuvant delivery to the tumor site can elicit significant DC maturation and an antitumor immune response. Zhou et al. demonstrated that the co-delivery of OVA antigen and R837 with PEGylated MnFe_2_O_4_ nanoparticles successfully induced *in vitro* and *in vivo* immune responses (Zhou et al., 2020a). Additionally, NIR irradiation comprehensively inhibited primary tumor growth and suppressed lung metastasis in an orthotopic breast cancer model (Zhou et al., 2020a). Chang et al. proposed multifunctional hollow mesoporous Cu_2_MoS_4_ (CMS) loaded with glucose oxidase (GOx) nanoparticles to modulate the TME for combined CDT, PTT, starvation therapy, and immune therapy (Chang et al., 2019). The hollow mesoporous CMS accommodated an enormous range of cavities to carry GOx, which catalyzed glucose to H_2_O_2_ to induce glucose starvation inside the tumor and regenerated H_2_O_2_ to assist the Cu and Mo ions in performing Fenton-like reactions. The presence of Cu^+^/Cu^2+^ and Mo^4+^/Mo^6+^ redox couples in CMS facilitated the significant production of hydroxyl radicals (•OH) to induce ferroptosis. The combination of NIR-irradiated cell death and ferroptosis-based cell death promoted TAA production and a robust immune response (Chang et al., 2019). Additional checkpoint blockade with anti-CTL antigen-4 (CTLA4) treatment effectively eliminated the primary and metastatic tumors (Chang et al., 2019). Therefore, the combination of immunotherapy with other therapeutic modalities has demonstrated a significant antitumor response, with robust immune activation against cancer cells for successful eradication without reoccurrence.

## Biosafety of Metallic Nanotherapeutics

The biosafety of metallic nanotherapeutics is an important aspect to assure that the toxic effects of these agents do not surpass their advantages during treatment. The toxicity of metallic nanotherapeutics is determined by their cellular and subcellular localization properties (Su et al., 2018). Moreover, the size of metallic nanotherapeutics is a key factor in determining cellular uptake. Metallic nanotherapeutics are transported through endocytosis and largely localized in lysosomes, cytoplasm, and the nucleus (Su et al., 2018). Bulk materials lack high cellular uptake and are easily cleared from the body by the liver (Su et al., 2018). Similarly, the shape of nanoparticles can play a critical role in cellular internalization (Xie et al., 2017). Lin et al. reported that triangle-shaped, rod-shaped, and star-shaped Au MNPs demonstrated different endocytosis rates (Xie et al., 2017). Triangle-shaped Au MNPs showed the highest cellular uptake, followed by rod-shaped, and then star-shaped Au MNPs (Xie et al., 2017). The toxicity of metallic nanotherapeutics depends on its biodegradability, the chemical meltability nature, and surface adsorption capacity. In the case of metallic nanotherapeutics, a larger surface per unit mass can induce the higher adsorption of proteins, nutrients, and growth factors, affecting the toxicity of the agent (Yao et al., 2019). However, the surface modification of metallic nanotherapeutics with biocompatible moieties can avoid unnecessary accumulation in different organs and protein adsorption, thereby reducing toxicity and side effects. Edward et al. reported that transferrin-modified Au nanoparticles demonstrated higher uptake in MDA-MB-435 cancer cells, whereas PEG-coated Au nanoparticles penetrated the tumors more deeply with a slower adsorption rate (Sykes et al., 2014). The surface modification of metallic nanotherapeutics enhances the biocompatibility of these agents by enabling an appropriate amount of absorption into the body without any side effects during treatment. MNPs tend to release metal ions when exposed to aqueous solutions or biological media (Yao et al., 2019). Furthermore, the toxicity of metallic nanotherapeutics depends upon the released metal ions, as well as their behavior and interaction with cellular organelles. The catalytic activities of metallic nanotherapeutics can induce ROS production, releasing a large number of metal ions inside the cells. Hence, the type of metallic nanotherapeutics, dosage, and its systemic behavior should be considered during treatment (Yao et al., 2019). Biosafety is an important aspect of nanotherapeutics, which needs to be prioritized during cancer therapy to reduce unnecessary side effects.

## Clinical Status

Ongoing and completed clinical trials have reported the application of MNPs in cancer therapy. Aurimune (CYT-6091) using 27-nm gold nanoparticles modified with thiolated PEG and recombinant human TNF-α (rhTNF-α) completed a phase I clinical trial in 2010 (Libutti et al., 2010). The delivery of rhTNF-α to cancer cells stimulated CD4+ and CD8+ cells and reduced regulatory T cell infiltration. PEGylated gold nanoparticles are an ideal candidate that successfully delivered rhTNF-α, and were cleared from the body after 120 days without any unnecessary damage to other tissues (Libutti et al., 2010). A silica-coated gold nanoshell, termed an AuroShell, was investigated in phase I clinical trials with 14 patients (Staves, 2010). For the treatment of head and neck cancer, a 120-nm silica nanoparticle with a 15-nm gold shell layer was modified with 5 kDa PEG for NIR laser (808 nm, 4 W/cm^2^)-irradiated PTT (Staves, 2010). AuroShell was administered intravenously at a dose of 24–31 mg/kg to the patients. Fourteen hours after treatment, the patient was exposed to an NIR laser (Staves, 2010). Following NIR irradiation, AuroShell treatment elevated the temperature and caused tissue damage within 6 min. AuroLase®, a nanoshell structed gold nano particle, was developed by Nano spectra biosciences company for photo thermal ablation of prostate cancer therapy. AuroLase® clinical trial has updated in August 2014 (NCT00848042) with 11 patients for Head and Neck cancer. In the follow-up trial, AuroLase® was investigated for thermal ablation of the targeted tumor with different doses like 4.5, 7.5 ml/kg, and different power. All the participants have experienced an adverse effect during the treatments and the study was continued for 6 months. Another clinical trial is active in the investigation of primary and metastatic lung cancer. AMF-based hyperthermia was investigated in phase I clinical trials in 2015 with an iron nanoparticle called Magnablate (NCT02033447). This study was conducted using 12 prostate cancer patients as an early phase I trial and the results are yet to be published (Staves, 2010). Various IONPs under phase I and phase II clinical trials with AMF induced magnetic hyperthermia owing to the favorable systemic tolerability of IONPs (Cortajarena et al., 2014). Ferumoxytol is a super paramagnetic iron oxide coated with carbohydrates and it is often used for iron deficiency anemia disease. Ferumoxytol is being investigated in various clinical trials for MR imaging of numerous cancers. One clinical trial is ongoing from 2015 with 90 patients for steady-state blood volume maps by using Ferumoxytol non-stoichiometric magnetite MR imaging in glioblastoma patients (NCT02359097). The primary objective of this trial is to investigate the steady-state cerebral blood volume (CBV) maps that are better than dynamic susceptibility contrast CBV maps in brain tumor blood vessel visualizations. The secondary objective of the trial is to quantitate CBV estimation, therapeutic response and survival assessment, correlation of relative CBV, and performance of Ferumoxytol administration in different stages of cancer. However, there is slow progress in metallic nanoparticle translation into the clinical stage. The significant challenge facing by MNPs from entering the clinical stage is insufficient optimization of physical properties to achieve maximum functionality. Multiple preclinical studies have been reported that by modulating the size and shape of MNPs can tune the therapeutic performances. These investigations should be carried out at the biological level to prove the widely adopted physical modifications of MNPs for successful anticancer therapy. Another major drawback is that many preclinical studies have been investigated in small animal models which complicate the accurate representation of therapeutic activity into the human model. Currently IONPs and GNPs are investigated for drug delivery and thermal ablation in cancer treatment. However, these MNPs are limited toward local treatment and solid tumors. The long-term toxicity of MNPs is another major concern because if these MNPs are not cleared through the kidney or their accumulation in different organs for a long time can causes severe side effects. So, these are the main concerns that obstruct the clinical progress of MNPs. Moreover, sufficient investigation is needed to understand the function of MNPs against our biological systems and how the physical modifications can impact its performances during preclinical stages to amplify future MNPs clinical translation.

## Future Perspectives and Conclusion

MNPs for cancer therapy have faced significant hurdles for FDA approval and very few of them have approved for clinical trials. There are several concerns about the MNPs such as toxicity, biodegradability, biodistribution inside the body, immunogenicity, and clearance from the body, that should be investigated more in detail before the clinical trials. Various MNPs have shown acute and chronic toxicities which limit their translation into clinical settings. Notably, MNPs synthesized biologically by using multiple biological sources such as plants and microbes might overcome this toxicity issue. Biosynthesized MNPs have shown less serum protein adsorption in the blood favorable for the *in vivo* stability. The size and shape of the MNPs affect the *in vivo* toxicity, pharmacokinetics, and clearance from the body. Extremely small MNPs can be easily cleared from the body through our excretion system where the larger MNPs can be accumulated in different major organs and further induce some systemic toxicity. So, the ideal size and shape of the MNPs are essential to determine the longer circulation and higher accumulation inside the tumor. MNPs exhibiting excellent catalytic activity like manganese, iron, copper, cerium, etc. can be degraded into ionic forms in the presence of intracellular stimuli like lower pH, GSH, and peroxidase and generate toxic radicals and ROS. Intracellular degradation of MNPs into ionic form can be cleared easily after the treatment, thereby it can reduce the long-term toxicity. Modification of MNPs with biocompatible polymers and biomolecules can affect systemic toxicity and cellular uptake. So, detailed and careful studies are required to understand the therapeutic potential of biocompatible MNPs. Multiple stimuli responsive MNP can be a suitable candidate to manipulate the TME for personalized anticancer therapy. Although the role of MNPs in tumor site accumulation, systemic toxicity, biodegradability, and systemic clearance are uncertain, the potential anticancer response demonstrated by the combination of stimuli and MNPs can be considered as a possible alternative in near future.

Developments in the field of nanomedicine, specifically metallic nanotherapeutics, present immense potential for cancer theranostics. In cancer nanomedicine, MNPs contribute to various functions, from treatment to imaging purposes, which can be easily made available for future therapeutic procedures. General requirements for designing potential MNPs are based on their size, shape, surface modifications, therapeutic competence, and toxicity profile. Based on the desired application, metal nanotherapeutics can be fine-tuned to the required size, shape, and morphology. Surface modification is essential to minimize systemic toxicity and active site accumulation. The higher surface area of metallic nanotherapeutics presents a great advantage and can be modified with biological molecules and ligands to strengthen its applicability toward treatment by targeting cancer cells and transporting multiple drugs. Small-sized MNPs can be easily degraded inside the body and cleared by our clearance system. The optical properties of metallic nanotherapeutics introduce a tremendous opportunity for *in vivo* imaging such as PA, CT, SERS, US, and MRI to track the MNPs delivery. Simultaneously, different stimuli can enhance the therapeutic ability of metallic nanotherapeutics. Multiple stimuli-triggered metallic nanotherapeutics, as well as their therapeutic activities, have been discussed in this review. Stimuli-triggering can alter the on-demand drug delivery at targeted sites, which can be controlled externally, a key point for advanced research. Internal stimuli-triggered metallic nanotherapeutics have been shown to modulate the TME by reducing hypoxic conditions and decreasing GSH concentrations to induce a favorable environment for treatment. External stimuli are superior options to modulate the activities of metallic nanotherapeutics to control treatment procedures. For example, light-triggered metallic nanotherapeutics can elevate the TME temperature, which can be controlled to induce ICD for enhanced immune therapy. Similarly, US-triggered metallic nanotherapeutics can penetrate deep tissues for ROS generation, which can be further investigated for clinical use. Currently, various IONPs and GNPs are used clinically for radiotherapy and hyperthermia but warrant advanced research to verify their therapeutic behavior, as well as the toxicity of metallic nanotherapeutics. However, various MNPs have demonstrated unwanted toxicities, and intensive research can overcome this barrier by modifying the size, shape, and surface functionalization with biocompatible molecules to generate a favorable therapeutic agent. Stimuli triggers can induce multiple therapeutic activities inside a tumor, a beneficial feature for therapy, and can control the drug dose concentrations. Although there are some limitations, metallic nanotherapeutics combined with stimuli-triggering can be developed as proficient therapeutic agents for anticancer therapy.

## Author Contributions

AM contributed to conceptualization, writing the manuscript, and drawing figures. SU and I-KP edited and revised the manuscript. All authors contributed to the article and approved the submitted version.

## Conflict of Interest

SU and I-KP declare that they are the topic editors of this special issue “Stimuli responsive nanoparticles for anti-cancer therapy.” The remaining author declares that the research was conducted in the absence of any commercial or financial relationships that could be construed as a potential conflict of interest.
